# Integrative genomic analyses combined with molecular dynamics simulations reveal the impact of deleterious mutations of Bcl-2 gene on the apoptotic machinery and implications in carcinogenesis

**DOI:** 10.3389/fgene.2024.1502152

**Published:** 2025-01-07

**Authors:** Ghazi Elamin, Zhichao Zhang, Depika Dwarka, Kabange Kasumbwe, John Mellem, Nompumelelo P. Mkhwanazi, Paradise Madlala, Mahmoud E. S. Soliman

**Affiliations:** ^1^ Molecular Bio-Computation and Drug Design Laboratory, School of Health Sciences, University of KwaZulu-Natal, Westville Campus, Durban, South Africa; ^2^ Department of Pharmaceutical Chemistry, College of Pharmacy, Karary University, Khartoum, Sudan; ^3^ School of Chemistry, Dalian University of Technology, Dalian, Liaoning, China; ^4^ Ezintsha, Faculty of Health Sciences, University of Witwatersrand, Johannesburg, South Africa; ^5^ Biotechnology and Food Science, Durban University of Technology, Durban, South Africa; ^6^ HIV Pathogenesis Programme, School of Laboratory Medicine and Medical Science, The Doris Duke Medical Research Institute, Nelson R. Mandela School of Medicine, University of KwaZulu-Natal, Durban, South Africa; ^7^ School of Laboratory Medicine and Medical Sciences, University of KwaZulu-Natal, Durban, South Africa

**Keywords:** Bcl-2, nsSNPs, mutations, genomic analyses, molecular dynamics simulations

## Abstract

**Objectives:**

Unlike other diseases, cancer is not just a genome disease but should broadly be viewed as a disease of the cellular machinery. Therefore, integrative multifaceted approaches are crucial to understanding the complex nature of cancer biology. Bcl-2 (B-cell lymphoma 2), encoded by the human Bcl-2 gene, is a critical anti-apoptotic protein that regulates cell death pathways, primarily by inhibiting apoptosis. It plays a pivotal role in maintaining cellular homeostasis by preventing premature or excessive cell death. Genetic variations and dysregulation of Bcl-2 are particularly significant in cancer, as they disrupt the normal apoptotic machinery, enabling cancer cells to evade programmed cell death. Single nucleotide polymorphisms (SNPs) are considered viable diagnostic and therapeutic biomarkers for various cancers. Therefore, this study explores the association between SNPs in Bcl-2 and the structural, functional, protein-protein interactions (PPIs), drug binding and dynamic characteristics.

**Methods:**

Comprehensive cross-validated bioinformatics tools and molecular dynamics (MD) simulations. Multiple sequence, genetic, structural and disease phenotype analyses were applied in this study.

**Results:**

Analysis revealed that out of 130 mutations, approximately 8.5% of these mutations were classified as pathogenic. Furthermore, two particular variants, namely, Bcl-2^G101V^ and Bcl-2^F104L^, were found to be the most deleterious across all analyses. Following 500 ns, MD simulations showed that these mutations caused a significant distortion in the protein conformational, protein-protein interactions (PPIs), and drug binding landscape compared to Bcl-2^WT^.

**Conclusion:**

Despite being a predictive study, the findings presented in this report would offer a perspective insight for further experimental investigation, rational drug design, and cancer gene therapy.

## 1 Introduction

The complex nature of cancer biology imposes a major challenge in cancer research and, consequently, the development of effective treatment regimes. The World Health Organization (WHO) estimated that 10 million patients globally died from various forms of cancer in 2020 alone. Many clinical trials do not provide significant success despite significant advances in diagnosis and innovative therapy methods (Karimi et al., 2022). Even though targeted therapy has been a successful approach in treating cancer, heterogeneous cancer still has a variety of clinical profiles and molecular alterations. Certain genetic alterations in cancer targets can make drugs more effective or, more often, cause them to become resistant to treatment (Jin et al., 2019). Drug resistance caused by mutations is a common occurrence in cancer. Thus, the mutation profile of patient malignancies plays a major role in determining the effectiveness of targeted therapy. Accurate molecular and genetic profiling of tumour cells is becoming a crucial step before implementing targeted therapy in patients (Jin et al., 2019).

Apoptosis, a programmed cell death process, is critical for maintaining cellular homeostasis and plays a pivotal role in preventing cancer development by eliminating defective cells. The oncoprotein B-cell lymphoma-2 (Bcl-2) family of proteins, comprising both pro-apoptotic and anti-apoptotic members, regulates the intrinsic pathway of apoptosis by controlling mitochondrial outer membrane permeabilization (MOMP) (Adams and Cory, 2017). Dysregulation of these proteins can lead to apoptosis evasion, a hallmark of cancer, highlighting their importance in oncogenesis and as potential therapeutic targets (Czabotar et al., 2014).

Apoptosis is mediated through two main pathways: the intrinsic (mitochondrial) pathway and the extrinsic (death receptor) pathway. The intrinsic pathway is regulated by the Bcl-2 family, where the balance between anti-apoptotic proteins (such as Bcl-2) and pro-apoptotic proteins (such as Bax and Bak) determines cell survival or death (Youle and Strasser, 2008). The extrinsic pathway, triggered by external signals, activates death receptors leading to caspase activation (Lavrik et al., 2005).

Recent research challenges the traditional view that the Bcl-2 family of proteins directly initiates cancer. Instead, current evidence suggests a more nuanced role where these proteins intervene in processes such as tetraploidization-dependent senescence, contributing indirectly to the cancerous phenotype (Barriuso et al., 2023). Tetraploidization, the process by which cells double their genome and typically undergo senescence to prevent malignant transformation, is intricately regulated by the Bcl-2 family. These proteins are key players in determining cell fate after tetraploidization, deciding between senescence and further steps leading to aneuploidy, a hallmark of cancer cells (Barriuso et al., 2023). Moreover, the initiation of carcinogenesis is increasingly understood to involve mechanisms that maintain unicellular genome integrity, such as DNA repair pathways and responses to hyperpolyploidy (Niculescu V, 2024; Niculescu V. F., 2024; Niculescu and Niculescu, 2024). These processes are crucial for cellular survival under stress but can also lead to genomic instabilities when dysregulated, setting the stage for cancer development (Chatterjee and Walker, 2017; Conway et al., 2024). The interplay between the Bcl-2 family proteins and these genomic maintenance mechanisms offers a potential explanation for their role in cancer beyond their traditional functions in apoptosis regulation. In light of these insights, the BCL-2 family’s influence on cancer appears linked with its impact on cellular responses to genome duplication errors and subsequent genomic instability. This perspective aligns with findings that implicate disrupted apoptosis pathways and aberrant cell survival signals in the broader context of cellular genome management, rather than direct oncogenic transformations.

Recent research has also expanded our understanding of apoptosis, revealing its more nuanced roles beyond the traditional pathway of programmed cell death. Particularly, the phenomenon of anastasis, the process by which cells recover from the brink of apoptosis, has garnered significant attention (Vasileva et al., 2024). Anastasis provides critical insights into cellular resilience and has profound implications for cancer therapy, where the ability of cells to evade death can contribute to treatment resistance (Mohammed et al., 2022). Anastasis not only challenges the finality traditionally associated with apoptosis but also highlights the “dark side” of apoptosis, where apoptotic processes contribute unexpectedly to cancer progression and other diseases (Zaitceva et al., 2021). This aspect of apoptosis, often referred to as its “dark side,” involves mechanisms where sub-lethal apoptotic signaling promotes adaptive capabilities in cells, potentially leading to enhanced metastatic properties and therapeutic resistance (Vasileva et al., 2024). The study of anastasis has revealed that cells can reverse death processes, a capability that was previously unrecognized. This reversal is not merely a return to homeostasis but often results in cells that acquire new properties or heightened survival strategies, which can include resistance to anti-cancer drugs. Understanding the signaling pathways involved in anastasis could open new avenues for cancer treatment, potentially leading to therapies that prevent the reversal of apoptosis in malignant cells (McDonald et al., 2021).

Incorporating the concepts of anastasis and the dark side of apoptosis into the current framework of cancer biology not only enhances our understanding of cellular death but also underscores the complexity of targeting apoptotic pathways in cancer therapy. These insights emphasize the need for a deeper investigation into the molecular mechanisms that govern these processes to devise more effective therapeutic strategies.

In recent years, Bcl-2 family proteins have been widely recognized for their central role in regulating apoptosis, which is critical in the context of cancer therapy. High Bcl-2 expression in solid tumors has often been associated with enhanced responsiveness to certain anticancer therapies. However, the role of Bcl-2 is not unequivocally beneficial, as the regulation of apoptosis in solid tumors presents both opportunities and challenges in clinical settings (Ichim and Tait, 2016; Kalkavan et al., 2023; Nano and Montell, 2024). While high Bcl-2 expression can render some tumor cells more susceptible to apoptosis induction, it can also contribute to therapy resistance by inhibiting the apoptotic pathways of specific chemotherapeutic agents or targeted therapies (Ichim and Tait, 2016; Kalkavan et al., 2023; Nano and Montell, 2024). These complexities highlight the need to carefully consider the dual role of Bcl-2 and the broader implications of apoptosis regulation in therapeutic strategies. Addressing these challenges requires an integrated understanding of apoptotic and non-apoptotic pathways and their interplay in solid tumors to optimize therapeutic outcomes.

Bcl-2 family proteins control apoptosis and are implicated in various tumour progressions (Rosser et al., 2003; Goff et al., 2013). This gene was the first to promote prolonged cell survival and growth rather than boost proliferation, demonstrating the importance of inhibiting cell death in tumorigenesis (Cory and Adams, 2002). Bcl-2 inhibits cytochrome c (cyt-c) release from the mitochondria, preventing caspases involved in apoptosis from activating (Yin et al., 1994). Bcl-2 overexpression or aberrant expression has been associated with many cancers’ emergence, progression, and relapse (Delbridge et al., 2016; Kitada et al., 2002). Consequently, Bcl-2 activity and protein levels have emerged as essential measures for determining the success or failure of clinical treatment and predicting patient outcomes (Delbridge et al., 2016). The sensitivity of malignant tumor cells to apoptosis can be efficiently boosted by either lowering Bcl-2 protein levels or suppressing Bcl-2 function (Qian et al., 2022). Multidrug resistance (MDR) in cancer cells can be overcome by selectively inhibiting Bcl-2, resulting in cell cycle arrest, senescence, and eventual cell death in response to radiotherapy and chemotherapy (Tang et al., 2020; Wang et al., 2020). Therefore, inhibition of Bcl-2 inactivation has become a highly attractive strategy in the battle against cancer, and BH3 mimetics are the main category of promising therapeutic agents (Perini et al., 2018; Delbridge and Strasser, 2015). BH3 mimetics inhibit Bcl-2 activity by competing with its physiological ligands, BH3 domain-containing pro-apoptotic proteins, at the hydrophobic (binding) groove (Czabotar et al., 2014).

Despite the promising initial clinical effectiveness of BH3 mimetic agents in various cancers, the mutation is a common way cancer cells evade therapies (Roberts et al., 2016; Stilgenbauer et al., 2018). The most common mutation is a change from glycine to valine at amino acid position 101 (G101V), which substantially decreases Bcl-2 affinity towards the BH3 mimetics agent (Venetoclax) and prevents the drug from displacing pro-apoptotic mediators from Bcl-2 in the cells (Blombery et al., 2019; Blombery et al., 2020). Most human genetic variations are attributable to single nucleotide polymorphisms (SNPs) (Dakal et al., 2017). This genetic variation generated by SNPs in genetic codons influences the translation outcome, resulting in a mutant protein with a different structure and function. Nevertheless, not all SNPs impact protein function and structure; a few are harmful, but many are not (Kucukkal et al., 2015).

Bioinformatics offers enormous array of databases and techniques that are necessary for the analysis, integration, and interpretation of cancer multi-omics data (Jiménez‐Santos et al., 2022). It is noteworthy that *in silico* techniques have recently emerged as valuable tool to assess the distinct genomic alterations and transcriptome profiles of tumors, as well as understanding the underlying mechanisms of cancer (Yalcin-Ozkat, 2021; Edelman et al., 2010; Elamin et al., 2024).

The primary goal of this study is to explore the effects of single nucleotide polymorphisms (SNPs) in the Bcl-2 gene on the structural, functional, and dynamic properties of the Bcl-2 protein. The specific objectives are to: identify and classify SNPs in the Bcl-2 gene and assess their pathogenic potential using bioinformatics and validated computational tools; analyze the structural and functional impacts of deleterious Bcl-2 mutations on protein-protein interactions (PPIs) and drug-binding characteristics; and pinpoint key mutations, such as Bcl-2^G101V^ and Bcl-2^F104L^, that significantly affect protein behavior, offering valuable insights for cancer diagnostics and therapeutic strategies. By identifying pathogenic SNPs and their effects on Bcl-2, the study enhances understanding of the molecular mechanisms underpinning apoptosis resistance in cancer. Moreover, the study highlights SNPs with diagnostic and therapeutic potential, offering a foundation for the development of biomarkers for cancer diagnosis and prognosis.

**FIGURE 1:**
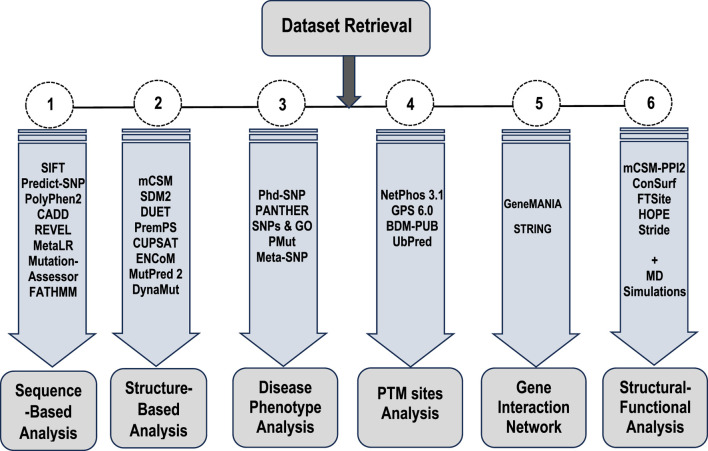
Flowchart of the different types of analyses and approaches employed in this study.

To achieve the objectives of this study, a combination of in silico methods, bioinformatics approaches, and molecular dynamics simulations was utilized to comprehensively investigate the genomic and proteomic alterations in Bcl-2 (Figure 1) and their potential roles in carcinogenesis. To ensure cross-validation and the reliability of the generated data, multiple bioinformatics algorithms were employed for each type of analysis conducted. Several mutations were examined for their potential contributions to cancer initiation and progression, with their deleterious effects on the structure and function of Bcl-2 thoroughly characterized. Subsequently, the most deleterious mutations, Bcl-2^G101V^ and Bcl-2^F104L^, were selected for further dynamic analysis to probe their impact on the protein conformational landscape using molecular dynamics (MD) simulations and post-dynamic analyses.

We believe that the extensive and multifaceted analyses provided in this study will offer a thorough grasp of the effects of deleterious Bcl-2 gene mutations on the apoptotic machinery and their implications for carcinogenesis. Our findings would contribute to the broader discourse on the genetic underpinnings of cancer, illustrating how specific SNPs can alter key apoptotic regulators such as Bcl-2. This understanding will then inform future directions in drug design and the development of anti-cancer therapeutics.

## 2 Methods

### 2.1 Generation of the datasets

The Bcl-2 FASTA sequence was obtained from UniProt (UniProt ID: P10415) (https://www.uniprot.org/) (Bateman et al., 2017). The dbSNP (https://www.ncbi.nlm.nih.gov/snp/) and Ensembl (https://www.ensembl.org/) databases and an extensive literature search were used to compile the list of mutations (Sherry et al., 2001; Hubbard et al., 2002). Gene synonyms (Bcl-2, PPP1R50) (transcript ID: ENST00000333681.5) of the Bcl-2 protein were selected for this study. Duplicate variants and other redundant data were excluded from the analysis. High-resolution crystal structures of the Bcl-2 protein, both wild-type and mutated (G101V and F104L) (PDB ID:6O0K, 6O0L, and 6O0M), were obtained from the Protein Data Bank (https://www.rcsb.org/) (Birkinshaw et al., 2019).

### 2.2 Sequence-based analyses for point mutation

We utilised eight different bioinformatics tools to obtain a reliable cross-validated sequence-based analysis to determine the deleterious effects of residue mutations on the protein. These are, the Sorting Intolerant From Tolerant (SIFT) algorithm (https://sift.bii.a-star.edu.sg) which determines the deleterious effects of residue mutations on proteins (Kumar et al., 2009); Polymorphism Phenotyping 2 (PolyPhen-2) (http://genetics.bwh.harvard.edu/pph2/) (Adzhubei et al., 2013), which is tailored to the study of high-throughput Next-Generation Sequencing (NGS) data and features multiple sequence alignments and classifiers based on machine learning; Combined Annotation Dependent Depletion (CADD) (https://cadd.gs.washington.edu/) that is designed to estimate the deleterious effect of residue variation on protein sequences (Rentzsch et al., 2019); Rare Exome Variant Ensemble Learner (REVEL) (https://sites.google.com/site/revelgenomics/) (Ioannidis et al., 2016); MetaLR (https://sites.google.com/site/jpopgen/dbNSFP) which predicts the deleteriousness of missense variants using logistic regression, which incorporates nine independent variant deleteriousness scores and allele frequency information (Liu et al., 2016); Mutation Assessor (http://mutationassessor.org/r3/) uses the evolutionary conservation of the impacted residues in protein homologs to speculate on the functional consequences of residue changes in proteins (Reva et al., 2011); Functional Analysis Through Hidden Markov Models (FATHMM) which is a high-throughput web server capable of predicting the functional consequences of both coding variants, that is, non-synonymous single nucleotide variants (nsSNVs) and non-coding variants in the human genome (http://fathmm.biocompute.org.uk/); and Predict-SNP (https://loschmidt.chemi.muni.cz/predictsnp1/) (Bendl et al., 2014).

### 2.3 Structure-based analyses for point mutation

Various algorithms were employed to predict the effect of missense mutations on the protein stability. These include, mCSM (https://biosig.lab.uq.edu.au/mcsm/) which uses various residues atomic distance patterns to train the predictive models (Pires et al., 2014a); Site-directed mutator2 (SDM2) (http://marid.bioc.cam.ac.uk/sdm2) which can also estimate the relative stability of wild-type and mutated protein structures by comparing them to known homologous 3D structures; DUET (http://biosig.unimelb.edu.au/duet/) which uses Support Vector Machines (SVM) to produce a consensual estimate (Pires et al., 2014b); PremPS (https://lilab.jysw.suda.edu.cn/research/PremPS/) which estimates changes in the Gibbs free energy of protein unfolding to assess the impact of single mutations on protein stability (Chen et al., 2020); CUPSAT (http://cupsat.tu-bs.de/) (Parthiban et al., 2006); ENCoM (https://labworm.com/tool/encom) (Frappier et al., 2015); MutPred2 (http://mutpred.mutdb.org/) (Pejaver et al., 2020); and DynaMut (https://biosig.lab.uq.edu.au/dynamut/) which takes the changes in vibrational entropy into account (Rodrigues et al., 2018).

### 2.4 Disease phenotype prediction analysis

Several machine learning and neural network algorithms were employed for disease phenotype prediction. These include, PhD-SNP (https://bio.tools/phd-snp) which uses neural networks that have been trained on a large library of standard and pathogenic mutations (Capriotti and Fariselli, 2017); Protein ANalysis THrough Evolutionary Relationships (PANTHER) (http://www.pantherdb.org/) which is designed to estimate the likelihood of a particular non-synonymous (residue changing) coding SNP that causes a functional impact on the protein (Thomas et al., 2022); SNPs and GO (https://snps.biofold.org/snps-and-go/) is another a precise technique that uses the associated protein functional annotation to determine whether or not a variation is associated with a disease based on a protein sequence (Capriotti et al., 2013); PMut (http://mmb.irbbarcelona.org/PMut/) which identifies pathogenic protein variants with up to 80% predictive accuracy in humans (López-Ferrando et al., 2017); and Meta-SNP (https://snps.biofold.org/meta-snp/) which is a randomised forest-based classification algorithm that distinguishes between polymorphic non-synonymous SNVs and disease-related one.

### 2.5 Post-transcriptional modification (PTM) sites prediction

PTM site predictions comprised several rearranged residues that produced many proteins. Ubiquitination, phosphorylation, and methylation are some of the PTM sites that have been characterised. These sites are essential in vital cellular organising processes such as pathological signaling cascades and protein-protein interactions. Thus, PTM prediction assisted in elucidating whether genetic variants were associated with or contributed to disease pathogenesis. We used four tools for this purpose, namely,; NetPhos 3.1 (https://services.healthtech.dtu.dk/service.php?NetPhos-3.1); Group-based Prediction System (GPS) 6.0 (http://gps.biocuckoo.cn/) (Xue et al., 2005); BDM-PUB (http://bdmpub.biocuckoo.org/) which is for protein ubiquitination site prediction using the Bayesian Discriminant Method; and UbPred (http://www.ubpred.org/).

### 2.6 Gene-gene interaction network analysis

The gene function can be better understood by studying the genes with which it interacts. The GeneMANIA and STRING databases were used to investigate the relationship between the Bcl-2 gene and other genes and to predict the effect of Bcl-2 nsSNPs on other associated genes. GeneMANIA (https://genemania.org/) is a database for identifying genes related to input genes using an extensive set of functional association data (Warde-Farley et al., 2010). These association data included co-expression, colocalisation, pathways, protein domain similarity, and interactions between proteins and genes. GeneMANIA can identify novel pathway members or complex members, genes missed during the screening process, or genes that perform a specific function, such as protein kinases. STRING (https://string-db.org/) is a database of both experimentally verified and theoretically predicted interactions between proteins (Szklarczyk et al., 2021). In STRING, and to ensure high reliability, “physical interactions” and “confidence” was set to 0.99. These interactions occur through computational prediction, inter-organism information transmission, and aggregation of interactions from other (primary) databases, and they can be either direct (physical) or indirect (functional).

### 2.7 Effect of point mutation on the structural and functional integrity of the protein

The formation of a protein complex is critical in controlling many biological activities. Therefore, different algorithms were employed to investigate the effect of Bcl-2^G101V^ and Bcl-2^F104L^ structural and functional properties. mCSM-PPI2 (http://biosig.unimelb.edu.au/mcsm_ppi2/) was used to predict the effects of missense mutations on protein-protein affinity (Rodrigues et al., 2019). mCSM-PPI2 uses graph-based structural signatures to model the effects of variations on the inter-residue interaction network, evolutionary information, complex network metrics, and energy terms to generate an optimised predictor. ConSurf (https://consurf.tau.ac.il/) is another tool we employed to estimate the evolutionary conservation of residue positions in a protein molecule based on the phylogenetic relationships between homologous sequences (Ashkenazy et al., 2016). The degree to which the residue position is evolutionarily conserved strongly depends on its structural and functional importance. The ConSurf value varied from 1 to 9, with one denoting residues with the least conservation and nine denoting residues with the most conservation. Other tools such as FTSite (https://ftsite.bu.edu/) (Ngan et al., 2012), HOPE (https://www3.cmbi.umcn.nl/hope/) and Stride (http://webclu.bio.wzw.tum.de/stride/) (Heinig and Frishman, 2004), were also used to provide deeper insight on the structural and functional integrity of the protein upon mutation.

### 2.8 Molecular dynamics (MD) simulations

#### 2.8.1 Systems preparation

The Protein Data Bank Repository (RCSB PDB) (https://www.rcsb.org/) provided a crystallized X-ray structure of the Bcl-2^WT^, Bcl-2^G101V^, and Bcl-2^F104L^ with PDB entries of 6O0K, 6O0L, and 6O0M, respectively. The water molecules in the crystal structure were removed, and the missing hydrogen atoms were substituted for them, with the correct charges assigned at neutral pH. The Schrödinger suite’s Protein Preparation Wizard was employed for initial structure processing and energy minimization. To further reduce steric clashes between residues, we used the OPLS-2005 force field to minimize energy while setting the RMSD threshold to 0.30 for all structures (Shivakumar et al., 2012).

#### 2.8.2 Molecular dynamics simulations and post-dynamic analysis

MD simulations were carried out using AMBER18 software and its Particle Mesh Ewald Molecular Dynamics (PMEMD) module (Case et al., 2024; Darden et al., 1993). Protein systems were modelled, and atomic charges were assigned state using the standard Amber (FF14SB) force field within the Amber package. An *in-house* pdb4amber script was used to modify, rename, and protonate (histidine) Bcl-2 (Maier et al., 2015). The LEAP module was employed to generate Bcl-2 parameters and topology files. This was also used for system neutralization. Molecular minimisation was carried out using a constraint potential of 500 kcal/mol, with partial minimisation for 2,500 steps and full minimization taking 5,000 steps. Furthermore, a gradual heating from 0 to 310 K was implemented in the system. The unconstrained equilibration was performed for 5 ns while the atmospheric pressure was maintained at 1 bar with the help of a Berendsen barostat (Berendsen et al., 1984). Subsequently, production stages were conducted over 500 ns to understand the structural consequences of the mutations on Bcl-2.

The enzyme coordinates of Bcl-2^WT^, Bcl-2^G101V^, and Bcl-2^F104L^ were saved every 1 ps, and their resultant trajectories were analysed using the AMBER18 integrated CPPTRAJ module (Roe and Cheatham, 2013). Post-MD analyses included root-mean-square deviation (RMSD), root-mean-square fluctuations (RMSF), radius of gyration (Rg), solvent accessible surface area (SASA), intramolecular hydrogen bonding, and dynamic cross-correlation matrix (DCCM). Furthermore, principal component analysis (PCA) was calculated to unravel the protein’s atomic displacement extent. The generated data and subsequent complexes were visualized using Microcal Origin analytical software (www.originlab.com), NMWiz implemented in Visual Molecular Dynamics (VMD) (https://www.ks.uiuc.edu/Research/vmd/) (Seifert, 2014; Humphrey et al., 1996).

## 3 Results

The Bcl-2 SNP dataset was obtained from the dbSNP and Ensembl databases. Approximately 52,619 variations in Bcl-2 have been identified, with 49,593 SNPs located in the intronic region, 163 SNPs classified as missense variants, 1,401 SNPs located in the 3′UTR area, 832 SNPs located in the 5′UTR region, and 115 synonymous variants, as reported by dbSNP and Ensembl. Missense mutations in the coding region were the current target of this study. As a result of further filtering to remove duplicate variations, 130 variants were selected for further investigation.

### 3.1 Sequence-based analysis of point mutation

**FIGURE 2:**
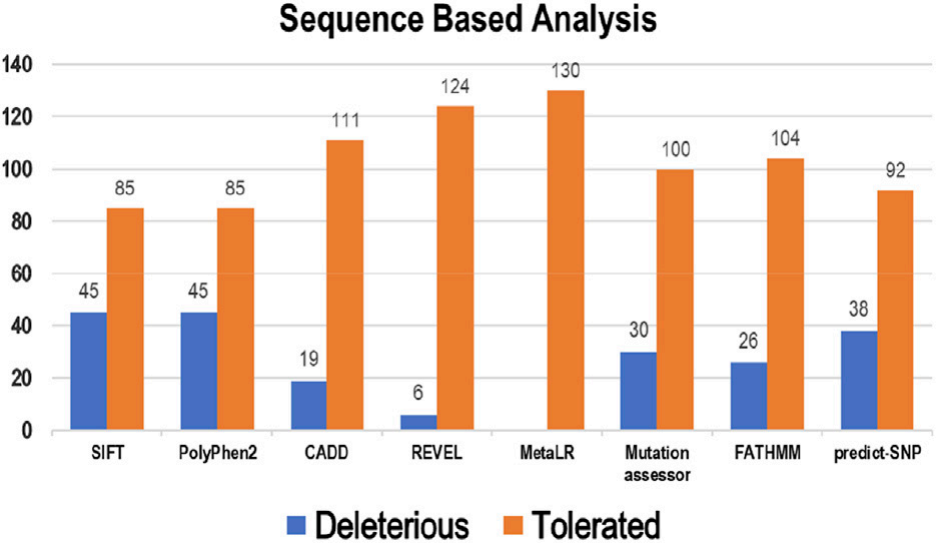
Deleterious and tolerated variations in Bcl-2 predicted through sequence-based algorithms.

Eight tools, namely, SIFT, PolyPhen2, CADD, REVEL, MetaLR, Mutation Assessor, FATHMM, and Predict-SNP were used to conduct sequence-based prediction and analyze the potential effects of Bcl-2 mutations. These eight tools separated deleterious mutations from tolerated ones (Supplementary Table S1). Out of 130 variants, SIFT and PolyPhen2 estimated 45 (∼35%) to be deleterious while CADD, REVEL, Mutation Assessor, FATHMM, and Predict-SNP predicted 19 (∼15%), 6 (∼5%), 30 (∼23%), 26 (∼20%), and 38 (∼29%) mutations as deleterious, respectively. However, the MetaLR algorithm predicted that all 130 (100%) variants were tolerated (Figure 2).

### 3.2 Structure-based analysis

**FIGURE 3:**
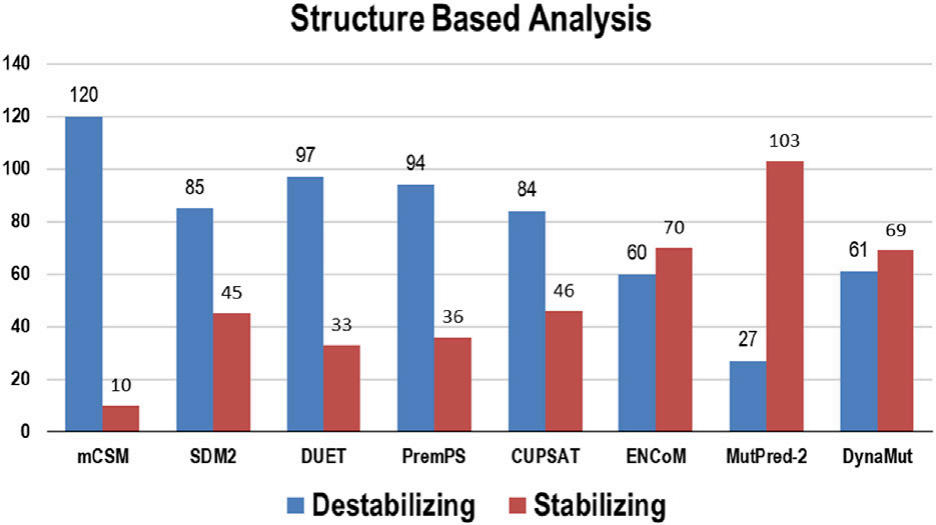
Destabilizing and stabilizing variations in Bcl-2 predicted through structure-based algorithms.

Multiple computational algorithms, including mCSM, SDM2, DUET, PremPS, CUPSAT, ENCoM, MutPred-2, and DynaMut were used to provide structure-based predictions of the effect of mutations. These tools distinguished between destabilizing and stabilizing mutations (Supplementary Table S2). The analysis concluded that out of 130 mutations, mCSM: 120 (∼92%), SDM2: 85 (∼65%), DUET: 97 (∼75%), PremPS: 94 (∼72%), CUPSAT: 84 (∼65%), ENCoM: 60 (∼46%), MutPred: 2–27 (∼21%), and DynaMut: 61 (∼47%) mutations were estimated to be destabilizing the structure of the protein (Figure 3).

### 3.3 Disease phenotype analysis

**FIGURE 4:**
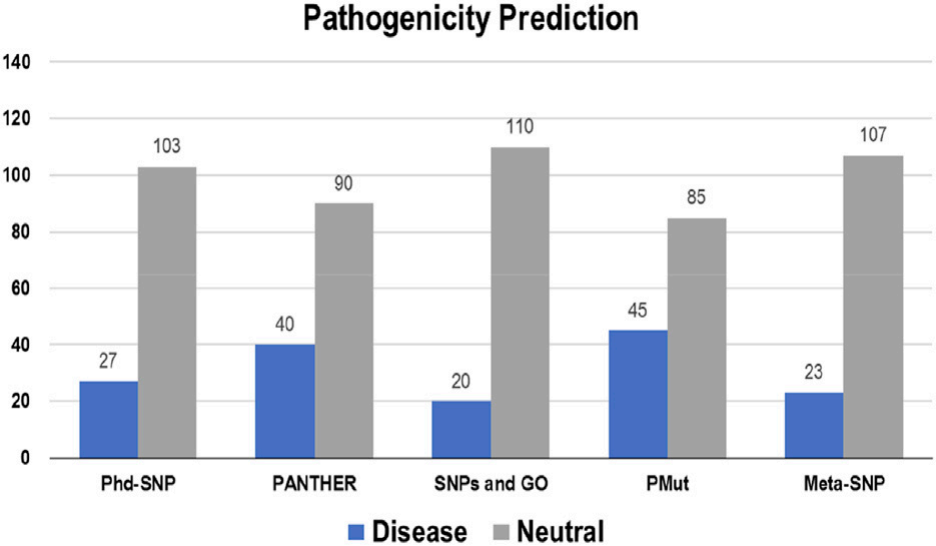
Disease and neutral variations in Bcl-2 predicted through disease phenotype prediction algorithms.

The pathogenicity of the targeted mutations was assessed utilizing PhD-SNP, PANTHER, SNPs and GO, PMut, and Meta-SNP. These algorithms use their prediction values to determine whether a specific mutation is disease-causing or neutral. From the 130 mutations, PhD-SNP predicted 27 (∼21%) mutations to be pathogenic, while PANTHER, SNPs and GO, PMut, and Meta-SNP predicted 40 (∼31%), 20 (∼15%), 45 (∼35%), and 23 (∼18%) mutations associated with the disease, respectively (Figure 4). However, only 11 of these mutations were predicted to be disease-causing across all the prediction algorithms (R12G, V15L, H94P, L97P, R98L, R129P, G141E, V142G, N143S, M166T, and G193R) (Supplementary Table S3).

### 3.4 Post-transcriptional modification (PTM) sites prediction

GPS-MSP 6.0 was used for methylation and determined the number of Bcl-2 sites that would be modified. However, GPS-MSP 6.0 predicted that phosphorylation would occur at 35 residues [Ser:15 (43%), Thr:12 (34%), and Tyr:8 (23%)]. In contrast, it was predicted by Netphose 3.1 those 20 different residues could be phosphorylated [Ser:11 (55%), Thr:7 (35%), and Tyr:2 (10%)].

Ubiquitination was predicted using BDMPUB and UbPred. BDMPUB anticipated that two lysine residues would be ubiquitinated, whereas UbPred projected those four lysine residues would be ubiquitinated.

### 3.5 Gene interaction network

**FIGURE 5:**
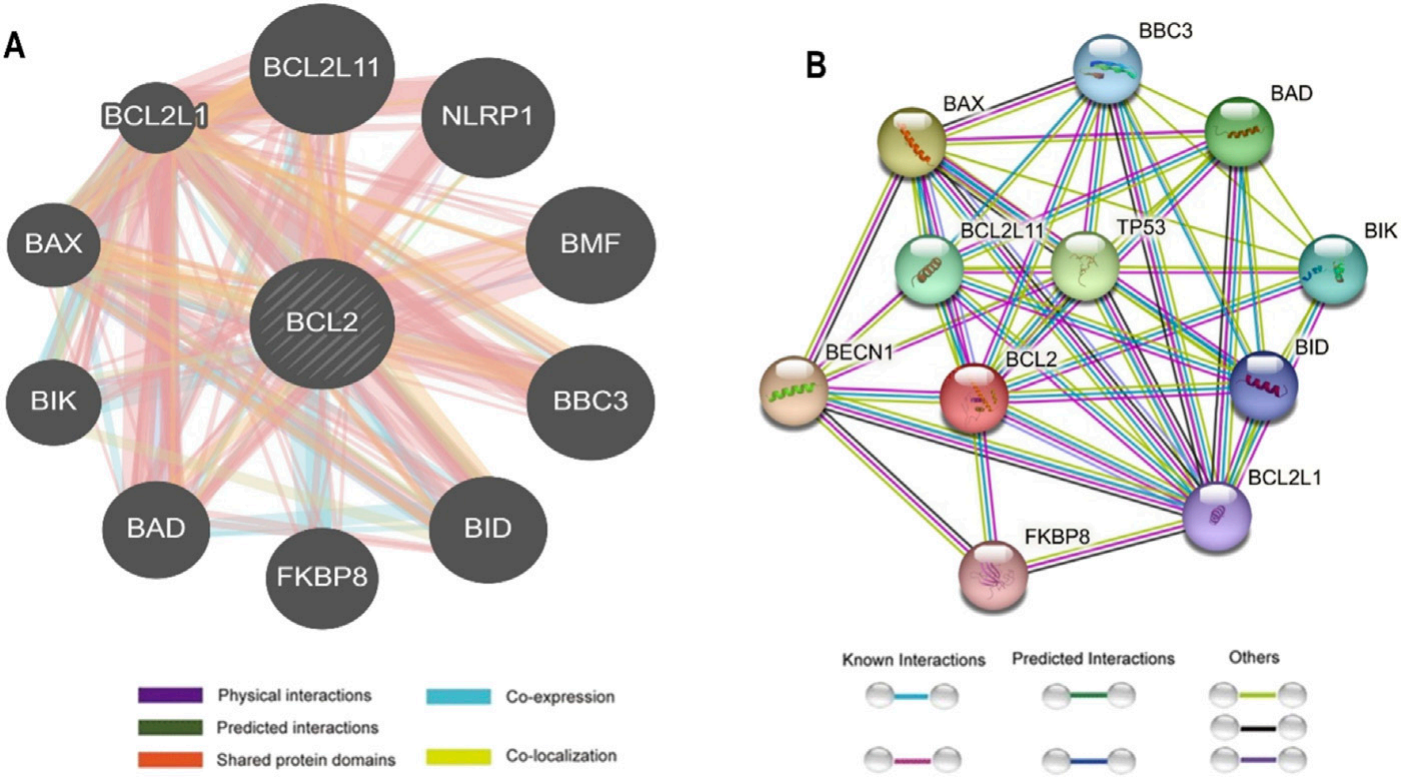
Bcl-2 Gene interactions with other genes predicted by **(A)** GeneMANIA and **(B)** STRING.

The interaction between Bcl-2 and other genes was evaluated using the GeneMANIA and STRING web servers. GeneMANIA analysis showed that Bcl-2 physically interacted with all ten genes and has no co-localization or genetic interaction with any other gene. However, Bcl-2 was co-expressed with BAX, BCL2L1, NLRP1, BBC3, and BID. Moreover, Bcl-2 shared protein domains with BCL2L1, BAX, BIK, and BID (Figure 5A).

The STRING database offers an integrated and comprehensive evaluation of indirect (functional) and direct (physical) protein-protein interactions. The network analysis revealed that Bcl-2 interacted directly 17 genes: BECN1, BAX, TP53, BAD, BCL2L11, BIK, BAK1, BBC3, BID, BCL2L1, HBK, BAG1, MCL1, APAF1, CREB1, NR4A1, and FKBP8 (Figure 5B).

### 3.6 Effect of mutations on the structural and functional integrity of Bcl-2

#### 3.6.1 Estimation of impact of mutation on protein-protein interactions (PPIs)

**FIGURE 6:**
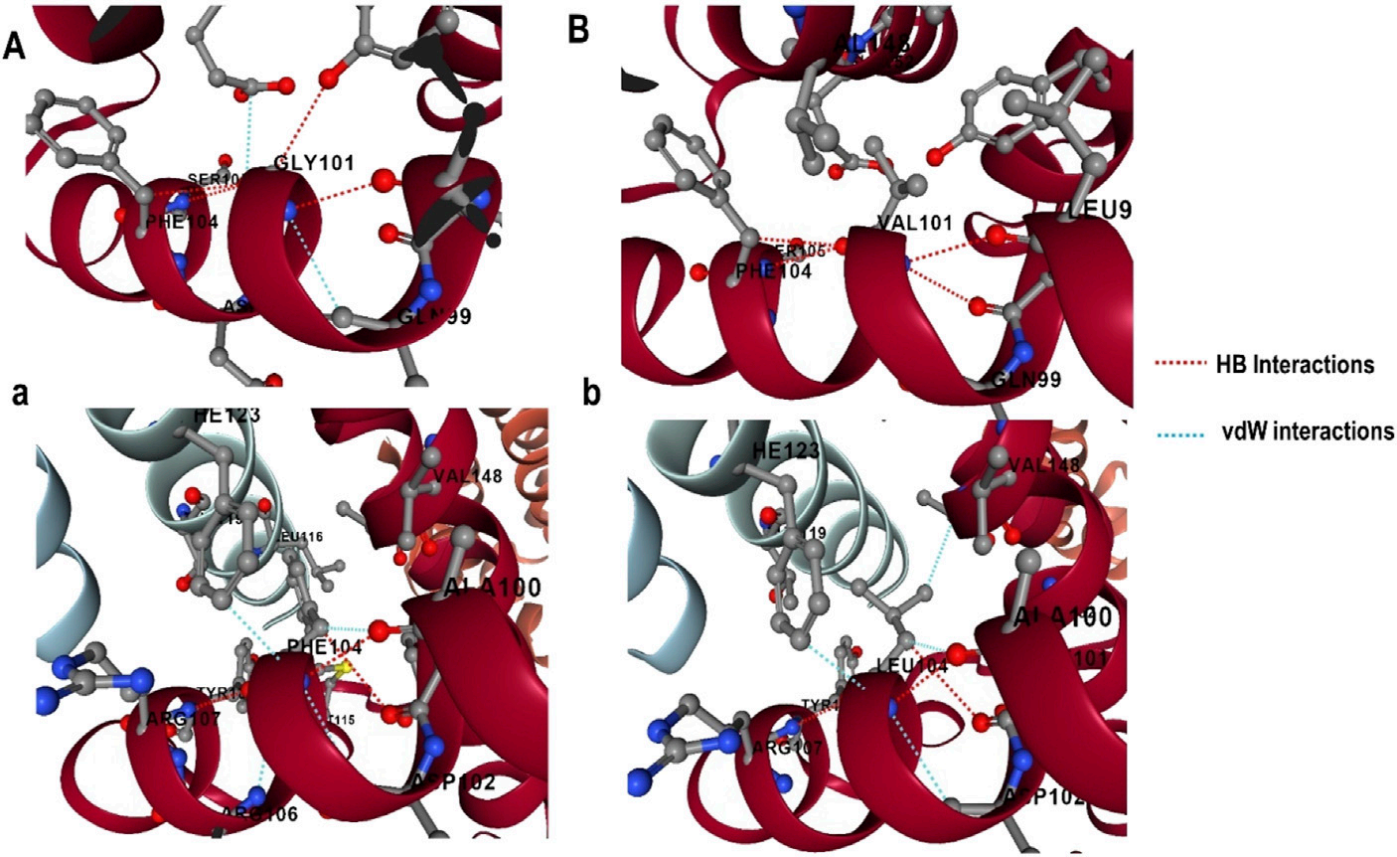
G101 and F104 residue interactions network of Bcl-2; **(A)** wild G101, **(A)** G101V variant, **(B)** wild F104, and **(B)** F104L variant as predicted by mCSM-PPI2.

The effect of mutations on the binding affinity of protein interactions was evaluated using mCSM-PPI2, which evaluates the effect of mutation by simulating the impact of variations on the network of non-covalent interactions between residues utilizing graph kernels, energetic terms, complex network metrics, and evolutionary data. The decreased binding affinity of protein-protein interaction was observed at the active site residues of the mCSM-PPI2-predicted Bcl-2 interaction, with a change in affinity (ΔΔ*G*_affinity_) of −0.559 kcal/mol for the G101V variant and −1.053 kcal/mol for the F104L variant. The interaction network revealed that the wild-type protein residue Gly101 established hydrogen bonds with Tyr18, Leu97, Arg98, Phe104, and Ser105, as well as van der Waals interactions with Gln99 and Glu152; however, in the mutant, Val101 established a hydrogen bond with Leu97, Arg98, Phe104, Ser105, and Glu152 (Figure 6). Likewise, the Phe104 in the wild-type generated hydrogen bonds with Ala100, Gly101, and Tyr108, and van der Waals interactions with Ala100, Asp102, Arg106, Tyr108, and Phe123, while in the mutant, leucine formed hydrogen bonds with the same residues in the wild-type (Figure 6).

#### 3.6.2 Conservation analysis of Bcl-2

**FIGURE 7:**
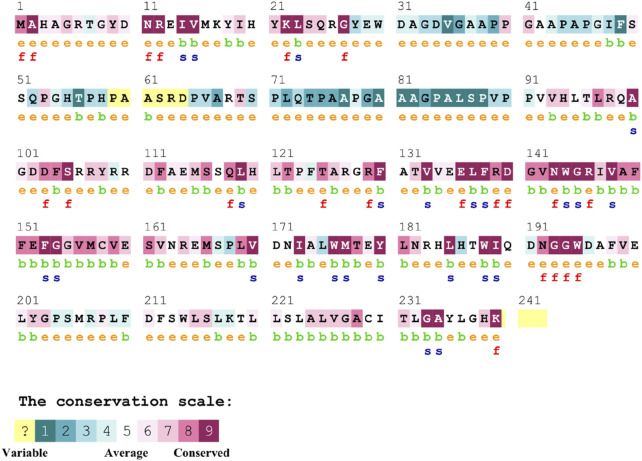
Sequence conservation plot of Bcl-2 protein generated using ConSurf web server.

The conservation of residues is the primary factor that ensures the structural integrity of proteins. The Bcl-2 structure’s conservation of residues was investigated using the ConSurf web server to comprehend its significance and localized evolution. The arrangement of residues and their degree of conservation was uncovered utilizing the ConSurf analysis. Several residues in Bcl-2 were shown to be relatively conserved using ConSurf, with particular emphasis on G101 and F104, suggesting that genetic variations at these positions might substantially impact Bcl-2 (Figure 7).

#### 3.6.3 Mapping ligand binding sites of Bcl-2

**FIGURE 8:**
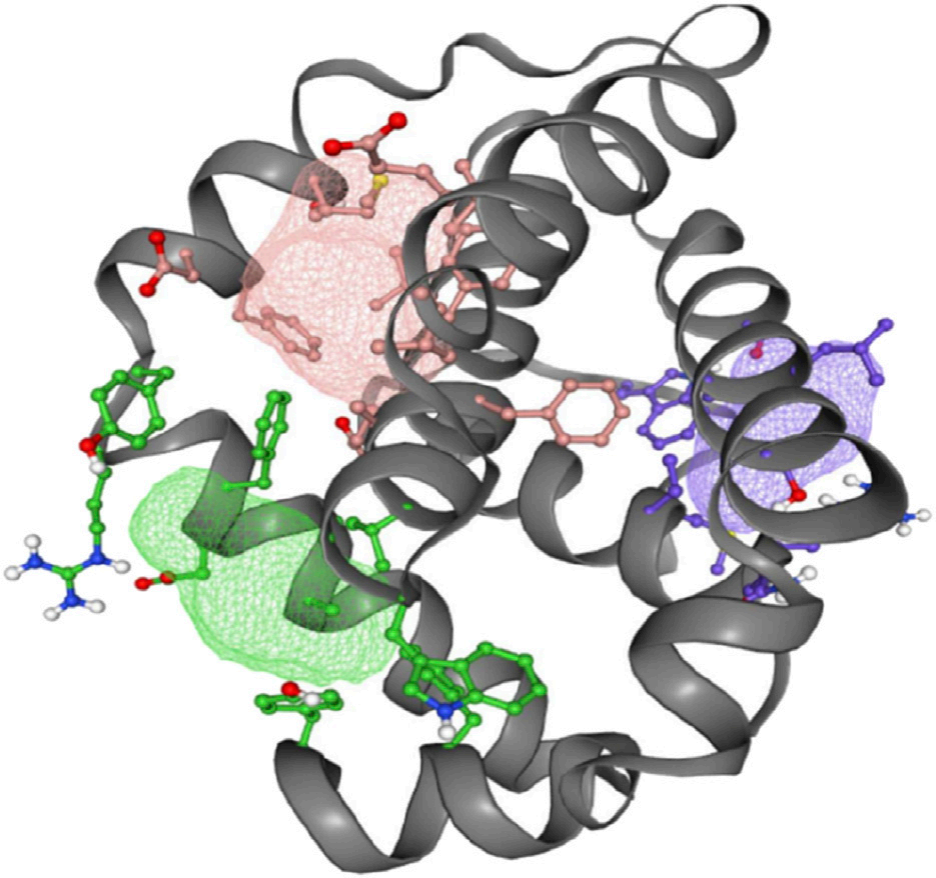
FT-site server prediction of the Bcl-2 protein ligand binding sites represented in mesh-like structure: pink (binding site 1), green (binding site 2), and purple (binding site 3).

The FT-site web server was used to identify Bcl-2 binding sites based on experimental evidence. The FT-site server depicted three ligand sites in Bcl-2. The ligand sites in Bcl-2 were represented by three different mesh-like structures on the FT-site server (pink, green, and purple), with corresponding residues that are within 5.0 Å of the binding site represented by ball and stick in these sites (Figure 8). The position of the F104 residue is detected in the first and second ligand-binding sites, while G101 is detected in the second ligand-binding site (Table 1). Consequently, mutations G101V and F104L may be more deleterious, as they potentially impact the Bcl-2 ligand-binding affinity.

**TABLE 1:** Bcl-2 protein ligand-binding sites and their respective residues.

Binding site 1 (pink)	Binding site 2 (green)	Binding site 3 (purple)
Phe104, Asp111, Phe112, Met115, Ser116, Val133, Glu136, Leu137, Ala149, Phe150, Glu152, Phe153, and Val156	Ala100, Gly101, Asp103, Phe104, Arg107, Tyr108, Trp144, Gly145, Val148, Phe198, and Tyr202	Asn11, Arg12, Val15, Met16, Trp30, Asp171, Ala174, Leu175, and Thr178

**FIGURE 9:**
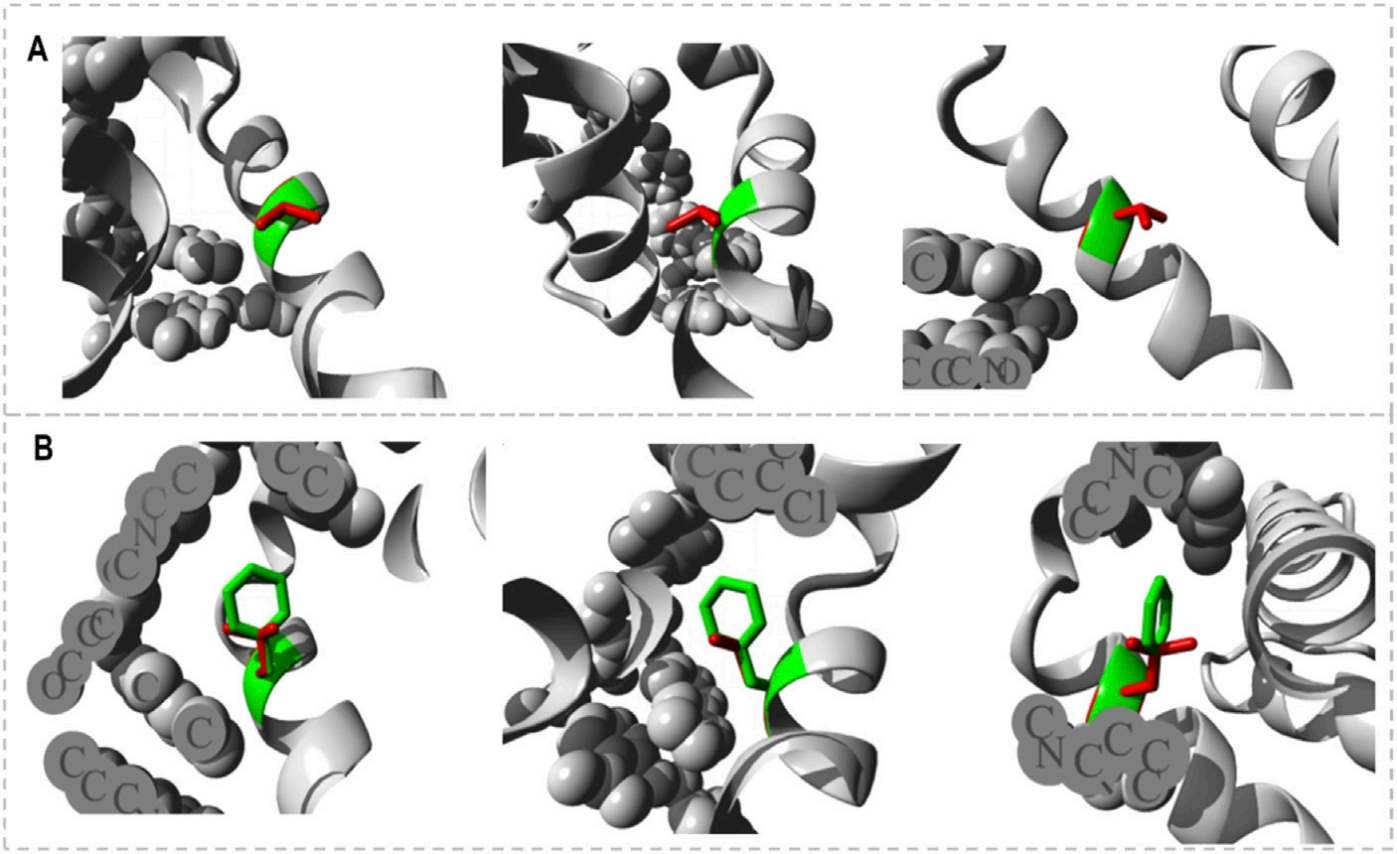
Close-ups (different angles) of the mutant and wild system; **(A)** Bcl-2^G101V^ and **(B)** Bcl-2^F104L^.

The HOPE project PDB viewer was used to visualize the structural features of the Bcl-2^WT^, Bcl-2^G101V^, and Bcl-2^F104L^ (Figure 9). Each residue demonstrated a unique size, charge, and hydrophobicity. These values frequently varied between the original wild-type and the newly introduced mutant residues. For the Bcl-2^G101V^, the mutant residue was bigger and more hydrophobic than the Bcl-2^WT^ residue. Although the mutated residue is not directly involved in ligand binding, it may indirectly affect ligand interactions made by other residues due to changes in local stability. The mutated residue is located within a special BH3 motif. Therefore, the different properties of residues caused the motif to become disrupted and consequently impair its function. Glycine had the highest degree of flexibility compared to other residues, which may be necessary for protein function. This function can be abolished by mutating this glycine. For Bcl-2^F104L^, the mutant residue was smaller than the Bcl-2^WT^ residue. The Bcl-2^WT^ residue interacted with Venetoclax, and the difference in properties between the Bcl-2^WT^ and mutant can easily cause a loss of interactions with the ligand. Protein function was frequently dependent on ligand binding, and this mutation may impair this function. The mutated residue was located within a special BH3 motif near a highly conserved position. Consequently, the motif was disturbed owing to the different properties of the residues, which would impede its function.

#### 3.6.4 Investigating the effect of the mutations on the protein secondary structure

**FIGURE 10:**
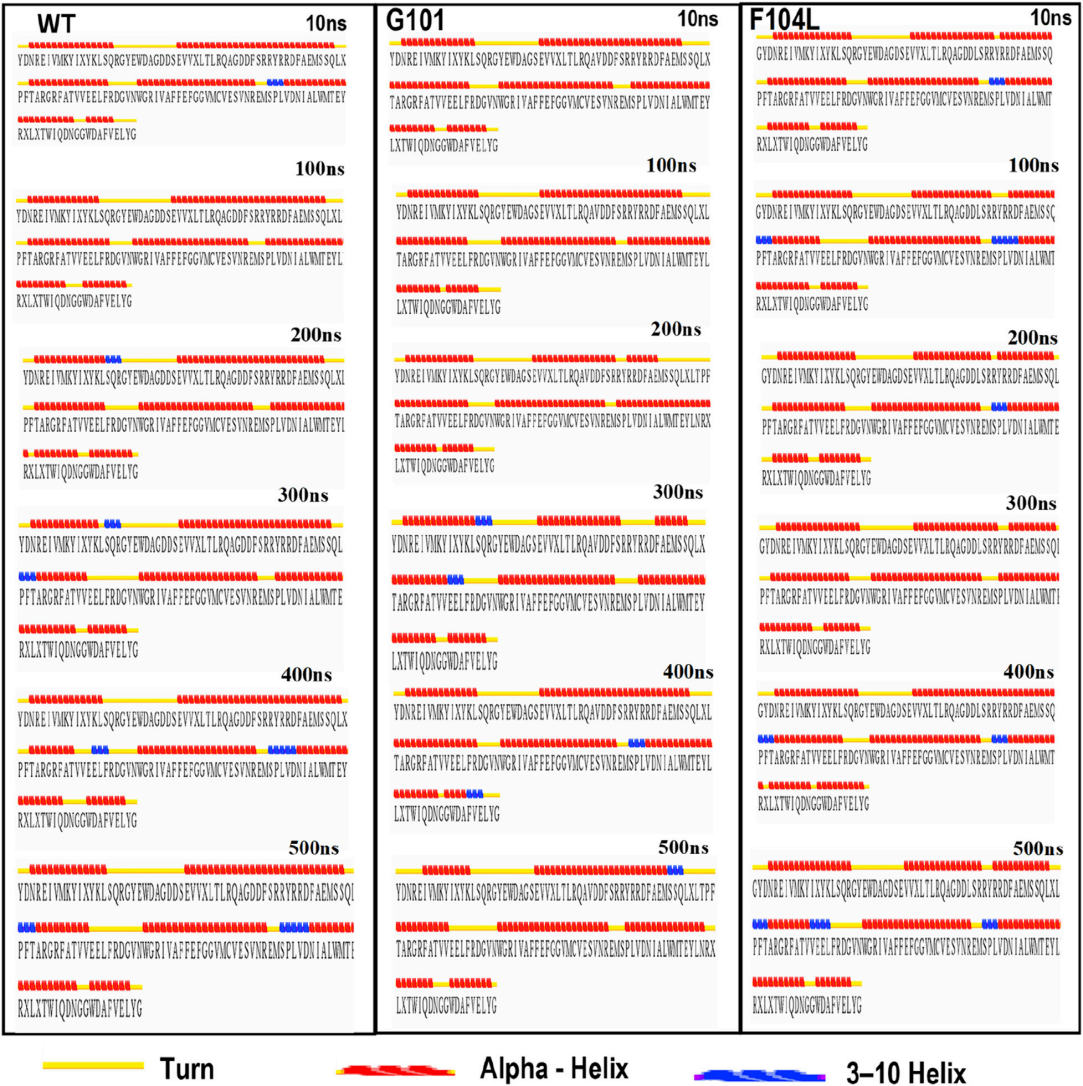
The secondary structural analysis of the Bcl-2WT, Bcl-2G101V, and Bcl-2F104L at 10, 100, 200, 300, 400, and 500 ns using the STRIDE web server.

MD trajectories of 500 ns were used to investigate the dynamics of secondary structural elements in Bcl-2^WT^, Bcl-2^G101V^, and Bcl-2^F104L^. This study contributed to a better understanding of the effects of genetic variations on the Bcl-2’s secondary structure through simulations. The STRIDE web server was used to detect the change in secondary structure at 10, 100, 200, 300, 400, and 500 ns (Figure 10). The secondary structural components in Bcl-2, such as *α*-helix, 3–10 helix, and turns, were divided into specific residues at each time interval. The Bcl-2^G101V^ and Bcl-2^F104L^ were observed to switch from a helix to a turn configuration at these residues.

### 3.7 Dynamic and conformational stability and fluctuations

**FIGURE 11:**
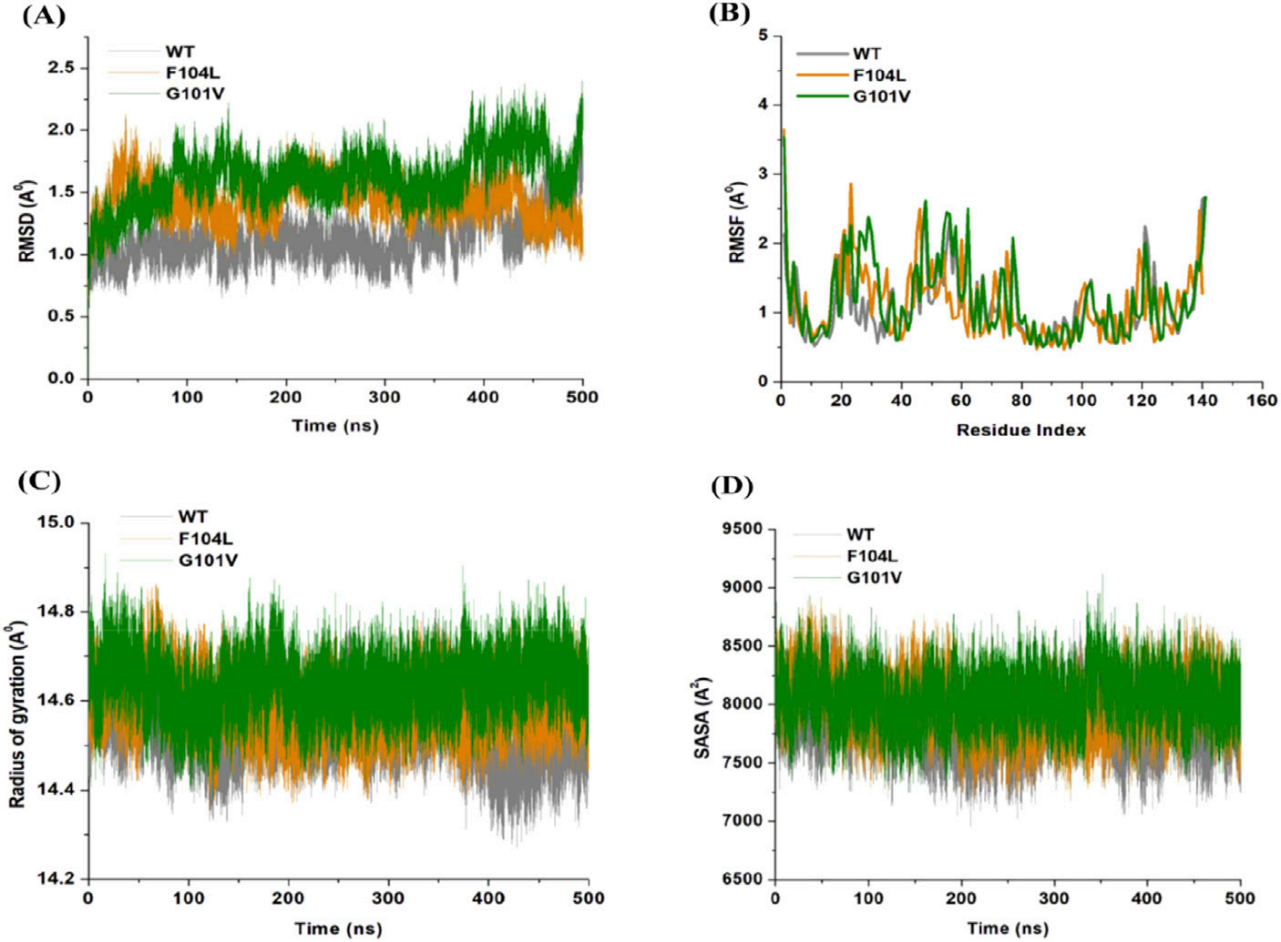
**(A)** RMSD, **(B)** RMSF, **(C)** Rg, and **(D)** SASA values across Cα of Bcl-2WT (gray), Bcl-2F104L (orange), and Bcl-2G101V (green) over 500 ns MD simulations.

The inherent behavior of a protein is associated with conformational changes and structural aberrations. Modifying a protein’s structure can significantly affect its function]. Therefore, understanding mutation-induced structural changes requires a more in-depth investigation of the conformational dynamics of proteins. For this reason, the effects of Bcl-2 mutations (G101V and F104L) were investigated over 500 ns MD simulations. The dynamics and stability of Bcl-2^WT^, Bcl-2^G101V^, and Bcl-2^F104L^ were determined by evaluating the time variable considering the RMSD of C_*α*_ atoms from computed trajectories. All systems reached convergence after 100 ns of the simulation period (Figure 11A). The Bcl-2^WT^ exhibited the lowest deviated RMSD value, 1.14 Å, while the Bcl-2^G101V^ and Bcl-2^F104L^ revealed higher RMSD values, 1.43 and 1.62 Å, respectively. The Bcl-2^G101V^ disrupted the RMSD pattern of Bcl-2^WT^ and caused it to fluctuate more than the Bcl-2^WT^ and Bcl-2^F104L^ during the simulation. The findings showed that Bcl-2^WT^ and Bcl-2^F104L^ displayed the least deviation of C_*α*_ atoms compared to Bcl-2^G101V^, indicating that the mutation of Gly to Val reduced the structural stability of Bcl-2. Furthermore, no significant variations in structural snaps were noticed, excluding the α3-α4 helices (hydrophobic groove) of superimposed Bcl-2^WT^, Bcl-2^G101V^, and Bcl-2^F104L^ every 100 ns during the simulation (Supplementary Figure S1). Here, *α*3-*α*4 helices become more dynamic and flexible as the simulation progresses, thus inducing expansion or shrinking in the hydrophobic groove, which appears most effectively in the Bcl-2^G101V^.

The relative rigidity and flexibility of residues determined protein conformational changes and their associated functions. Consequently, the RMSF values of Bcl-2^WT^, Bcl-2^G101V^, and Bcl-2^F104L^ can be computed and analyzed to see how Bcl-2’s residual fluctuations change due to mutations (Figure 11B). Bcl-2^WT^ demonstrated the least fluctuations of the residues with an average RMSF value of 1.10 Å when compared to 1.13 and 1.20 Å for the Bcl-2^F104L^ and Bcl-2^G101V^, respectively. The calculated trajectory showed a slightly higher pattern of fluctuations, especially for the Bcl-2^G101V^ variant. As a result of these mutations, the regions surrounding the various sites become more dynamic and internally disturbed, reflecting higher fluctuations in Bcl-2. The RMSF distribution correlated with the RMSD pattern, with mutated systems exhibiting more significant fluctuations. The substantial variations in the mutants’ residual fluctuations could be attributed to Bcl-2 structural inactivation.

Furthermore, the Rg values of all three systems were analyzed to determine the folding behavior and overall conformational changes in the Bcl-2 structure before and after mutation induction. The compactness, stability, and folding of a protein can be determined from the change in Rg values over time. The Rg values of the Bcl-2^WT^, Bcl-2^G101V^, and Bcl-2^F104L^ were estimated from the MD trajectories and plotted (Figure 11C). Bcl-2^WT^ had the lowest Rg value (14.54 Å, while the Bcl-2^F104L^ and Bcl-2^G101V^ showed slight increases at 14.59 and 14.63 Å, respectively. Altogether, Rg analysis of Bcl-2 revealed that the mutants were less stable, more flexible, and less compact than the native protein.

Moreover, the Bcl-2 structure’s hydrophilic and hydrophobic residues were analyzed using SASA. The SASA values for the Bcl-2^WT^, Bcl-2^G101V^, and Bcl-2^F104L^ were obtained and plotted throughout the 500 ns of MD simulation (Figure 11D). Following exposing the system to the solvent, Bcl-2^WT^ had a median SASA value of 7,824 Å^2^. The Bcl-2^G101V^ exhibited a higher SASA value of 8,049 Å^2^ than that of the Bcl-2^F104L^, which displayed a value of 7,985 Å^2^. The SASA values of all three systems agreed with the Rg results. The differences in the SASA values for the three systems throughout the simulation reflect Bcl-2 unfolding and folding. The overall SASA values for Bcl-2^WT^ and Bcl-2^F104L^ were slightly different, suggesting that the structural mutation from Phenylalanine to Leucine at position 104 in Bcl-2 provides better exposure to solvent compared with Bcl-2^G101V^ and, thus, favors the enhanced activity of the Bcl-2^F104L^ over that of the Bcl-2^G101V^.

#### 3.7.1 Hydrogen bonding analysis

**FIGURE 12:**
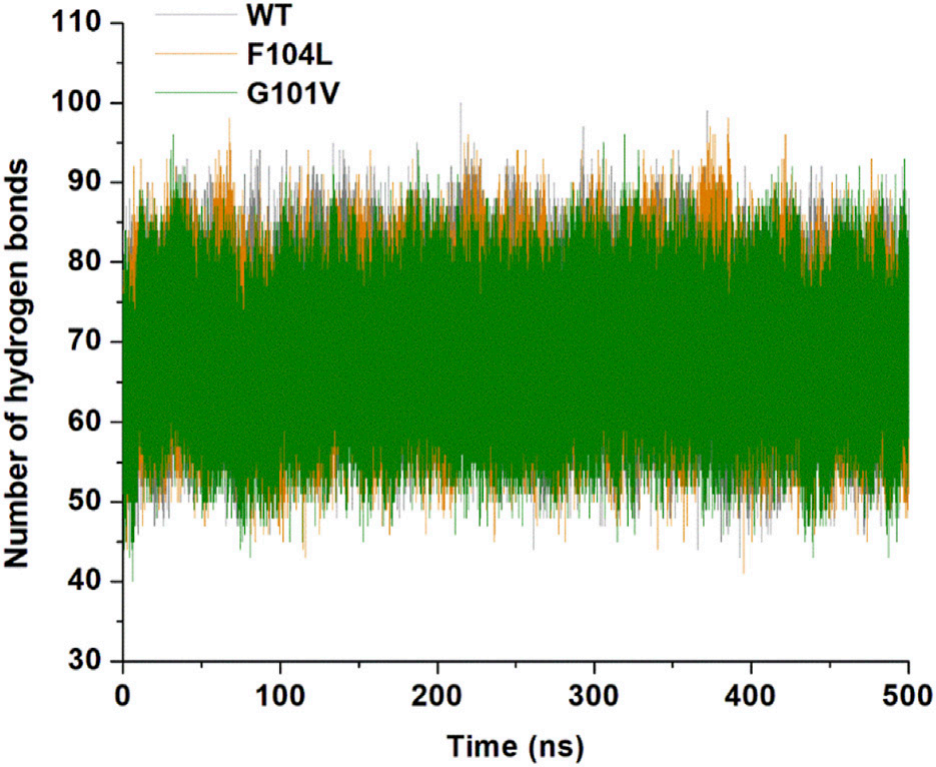
Intramolecular hydrogen bonding in Bcl-2WT (gray), Bcl-2F104L (orange), and Bcl-2G101V (green) over 500 ns MD simulations.

Analysis of intramolecular hydrogen bonds primarily assists in evaluating the overall conformation and stability of the protein structure through MD simulations. Time-dependent intramolecular hydrogen bond analysis was performed and plotted to evaluate the effect of mutations on the structure of Bcl-2 (Figure 12). The average values of intramolecular hydrogen bonds in Bcl-2^WT^, Bcl-2^G101V^, and Bcl-2^F104L^ ranged from about (43–100), (41–98), and (40–96), respectively, indicating a slight change before and after mutation formation. The Bcl-2^F104L^ and Bcl-2^WT^ models were more compact and stable than the Bcl-2^G101V^ model, and the results maintained a roughly similar trajectory pattern.

#### 3.7.2 Dynamic cross-correlation matrix (DCCM)

**FIGURE 13:**
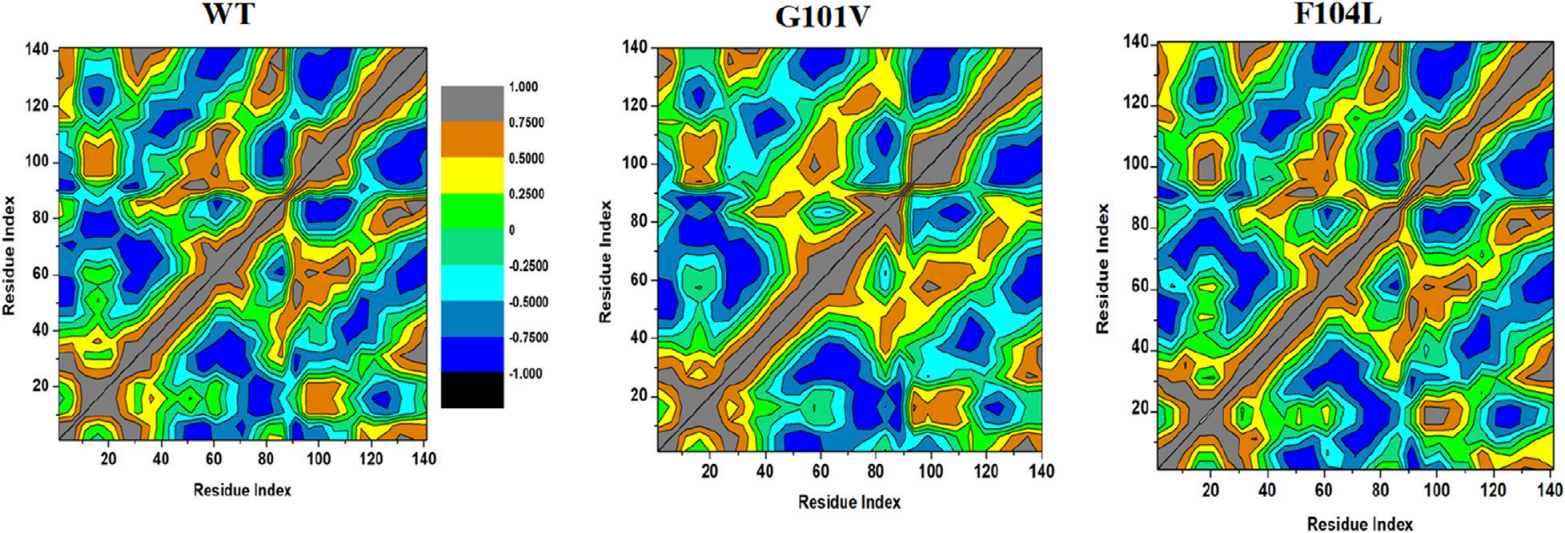
DCCM analyses for Bcl-2^WT^, Bcl-2^G101V^, and Bcl-2^F104L^ over 500 ns MD simulations.

To examine the differences in the dynamics of Bcl-2^WT^, Bcl-2^G101V^, and Bcl-2^F104L^, DCCM plots were generated for anti-correlated and correlated protein structural motions. The residues’ motion values range from −1 to +1. Positive values indicate positively correlated motions (brown colour), whereas negative values indicate anticorrelated motions (black colour) between residues (Figure 13). The scatter plots revealed that motion modes between residues of Bcl-2^F104L^ are similar to those of Bcl-2^WT^, whereas the Bcl-2^G101V^ showed a slightly different pattern, mutation obviously enhances the positively correlated motions occurring in the Bcl-2.

#### 3.7.3 Principal component analysis (PCA)

**FIGURE 14:**
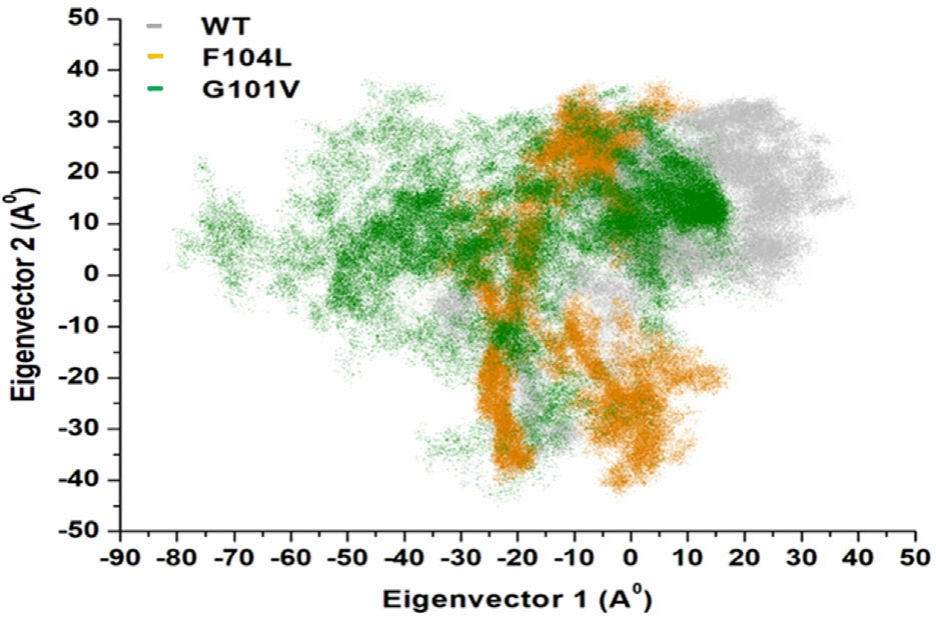
PCA for Bcl-2^WT^ (gray), Bcl-2^F104L^ (orange), and Bcl-2^G101V^ (green) over the 500ns MD simulations.

Intensive movements in Bcl-2^WT^, Bcl-2^G101V^, and Bcl-2^F104L^ were evaluated using PC analysis with the first two eigenvectors (EVs) to qualitatively examine the influence of induced mutations on the major conformational movements of each residue (Kumalo et al., 2016). The eigenvectors illustrate the directions of Bcl-2 motion, and the eigenvalues represent the overall motion strength; these are obtained by diagonalizing the covariance matrix (Chen et al., 2021; Chen et al., 2022). The conformational changes of Bcl-2 and its variants were shown in a 2D scatter plot (Figure 14), indicating a significant change in Bcl-2 overall movements after acquiring the mutations, especially Bcl-2^G101V^. Moreover, Figure 14 shows that the Bcl-2^G101V^ and Bcl-2^F104L^ with the trace covariance matric of 12.46 and 22.46 Å^2^, respectively, imposed highly fluctuated anti-correlated effects as the negative values of 2D scatter point into the protein. In the case of Bcl-2^WT^, the trace covariance matrices were 24.09 Å^2^, indicating the presence of prominent correlated motions with minimal system fluctuations. Consequently, the findings demonstrated that the Bcl-2^G101V^ caused substantial fluctuations in the simulated Bcl-2 dynamics.

## 4 Discussions

### 4.1 Sequence, structure, phenotype-mutational analysis and gene interactions

To ascertain the deleterious effect of residue mutation on the protein, we employed various sequence-based point mutation algorithms. Out of 130 mutations, SIFT and PolyPhen2 algorithms displayed the highest estimation, deeming 45 mutations (∼35%) deleterious. With the exception of the MetaLR algorithm, which predicted that all 130 (100%) variants were tolerated, other algorithms displayed results ranging from around 5 to 23 percent (Figure 2). We hypothesize that the inclusion of machine learning and high-throughput Next-Generation Sequencing (NGS) data in the PolyPhen2 method broadened the search field, contributing to the high prediction rate. Similarly, various algorithms were adopted to predict the effect of missense mutations on the protein stability, and to distinguish between destabilizing and stabilizing mutations. Out of 130 mutations, 3 algorithms (ENCoM, MutPred, and DynaMut) assessed between 21% and 46% of mutations are destabilizing, while 4 predictive tools (mCSM, SDM2, DUET, PremPS, and CUPSAT) estimated between 65% and 92% of mutations are destabilizing (Figure 3). We believe that the analysis adopted here is robust and reliable as we opted to combine various algorithms that take into account critical structural features such as protein folding and Gibbs’s free energy (PremPS), site-directed mutations relative to wild type (SDM2), vibrational entropy (DynaMut) and consensual estimation (DUET). A number of machine learning and neural network techniques were used to predict disease phenotypes (Figure 4; Supplementary Table S3), yet only 11 mutations were shown to be disease-causing by all prediction algorithms. These mutations are R12G, V15L, H94P, L97P, R98L, R129P, G141E, V142G, N143S, M166T, and G193R. GeneMANIA and STRING database offer an integrated and comprehensive evaluation of indirect (functional) and direct (physical) protein-protein interactions. The network analysis revealed that Bcl-2, Bcl-2 shared protein domains with BCL2L1, BAX, BIK, and BID (Figure 5A), and interacted directly with BECN1, BAX, TP53, BAD, BCL2L11, BIK, BAK1, BBC3, BID, BCL2L1, HBK, BAG1, MCL1, APAF1, CREB1, NR4A1, and FKBP8 (Figure 5).

### 4.2 Impact of mutations on protein-protein interactions

To explore the impact of Bcl-2^G101V^ and Bcl-2^F104L^ on their structural and functional characteristics, we utilized various techniques. The mCSM-PPI2 algorithm predicted a reduction in the binding affinity of protein-protein interaction, G101V variant change affinity (ΔΔ*G*_affinity_) with −0.559 kcal/mol, compared with −1.053 kcal/mol for F104L variant. According to the interaction network, Gly101in the wild-type protein, generated hydrogen bonds with Tyr18, Leu97, Arg98, Phe104, and Ser105, and exhibited van der Waals interactions with Gln99 and Glu152. However, in the mutant, Val101 established hydrogen bonds with Leu97, Arg98, Phe104, Ser105, and Glu152 (Figure 6). Furthermore, in the wild-type, Phe104 established hydrogen bonds with Ala100, Gly101, and Tyr108 and van der Waals interactions with Ala100, Asp102, Arg106, Tyr108, and Phe123. While in the mutant, leucine established hydrogen bonds with the same residues (Figure 6). The ConSurf web server was utilized to confirm the structural integrity of the Bcl-2 protein. Several residues in the Bcl-2 protein were shown to be relatively conserved, with a specific focus on G101 and F104., suggesting that genetic variations at these positions might substantially impact Bcl-2 (Figure 7). Additionally, FTSite, HOPE, and Stride were employed to gain further understanding of the structural and functional integrity of the Bcl-2 protein following mutation. The FT-site server depicted three ligand sites in Bcl-2 (Figure 8). According to Table 1, the first and second ligand-binding sites detect the position of the F104 residue, while the second ligand-binding site detects the G101 residue. Considering they may affect the Bcl-2 ligand-binding affinity, mutations G101V and F104L may thus be more deleterious. The Bcl-2^WT^, Bcl-2^G101V^, and Bcl-2^F104L^ structural characteristics were visualized using the HOPE project PDB viewer (Figure 9). The Bcl-2^G101V^ mutant residue exhibited a bigger size and greater hydrophobicity compared to the Bcl-2^WT^ residue. The mutant residue of Bcl-2^F104L^ was smaller than the residue of Bcl-2^WT^. Venetoclax was bound to the Bcl-2^WT^ residue, and because the two amino acids had different characteristics, the mutant form of Bcl-2^WT^ can readily lose its binding affinity for the ligand. Finally, the STRIDE web server was utilized to identify alterations in the secondary structure at specific time points: 10, 100, 200, 300, 400, and 500 ns (Figure 10). The conformational switch from a helix to a turn was seen in Bcl-2^G101V^ and Bcl-2^F104L^ at these residues.

### 4.3 Effect of mutations on the structural and dynamic landscape of the protein

We employed the MD simulations to conduct a comprehensive analysis of the conformational dynamics of proteins to understand the structural alterations caused by mutations. These mutations affected Bcl-2’s stability, flexibility, solvent-accessible surface area, and rigidity, as demonstrated by 500 ns MD simulations (Figure 11). Moreover, mutations impacted Bcl2’s hydrogen bond formation, and the Bcl-2^F104L^ and Bcl-2^WT^ models exhibited greater compactness and stability compared to the Bcl-2^G101V^ model (Figure 12). To explore mutation-induced effect on conformational alterations of Bcl-2, DCCMs and PCA are estimated. The results showed that the Bcl-2^G101V^ mutation clearly affects the positively correlated motions occurring in the Bcl-2 and causes substantial fluctuations in the simulated Bcl-2 dynamics (Figures 13, 14).

Overall, the findings of this study hold several biological significances, for instance having information on SNPs in the Bcl-2 gene would help identify potential biomarkers for cancer diagnosis and treatment. Furthermore, by examining the structural and functional effects of SNPs in Bcl-2, our finding may pinpoint novel targets for cancer therapy. Treatments that specifically target genetic variants or protein interactions linked to Bcl-2 SNPs may be able to return cancer cells to normal apoptotic pathways, which would ultimately result in their elimination. Information presented here on how SNPs in Bcl-2 influence protein-protein interactions can provide insights into the molecular mechanisms underlying cancer development and progression.

## 5 Limitation of the study and future perspective

The current study has focused exclusively on the association between single nucleotide polymorphisms (SNPs) in the Bcl-2 gene and various aspects of its function and interactions in human cells. One limitation of this approach is the exclusion of comparative genomic analyses, which could provide additional insights into the evolutionary history and functional differences between Bcl-2 in cancerous versus non-cancerous cells, as well as comparisons with unicellular organisms such as yeasts. Such comparisons might reveal conserved or divergent evolutionary traits that contribute to our understanding of Bcl-2 role in apoptosis and oncogenesis across different species and cell types. Moreover, the study does not address the broader genomic context that may influence these variations, such as regulatory elements or interactions with other genomic regions. The potential impact of epigenetic factors on Bcl-2 expression and function is also not explored, which could be significant given the gene’s role in critical cellular processes.

Future studies could expand on the current work by incorporating comparative genomics to analyze Bcl-2 across different species, including model organisms like yeasts, which can offer valuable insights due to their simpler genetic backgrounds and ease of genetic manipulation. Such studies would enhance our understanding of the evolutionary pressures that have shaped the Bcl-2 gene and could identify conserved elements critical for its function. Additionally, examining the interplay between Bcl-2 SNPs and other genomic or epigenetic factors could provide a more comprehensive picture of how Bcl-2 variants contribute to disease phenotypes. This could involve integrating broader genomic data sets, including whole-genome sequencing and epigenetic profiling, to discern the complex regulatory networks that impact BCL-2 expression and activity. In light of these limitations, further research could also focus on the translational application of our findings, exploring how the SNPs identified could influence the efficacy of BCL-2-targeted therapies in clinical settings.

By addressing these areas, future research could provide a deeper understanding of BCL-2’s role in disease and offer new avenues for therapeutic intervention, ultimately leading to improved treatment strategies for diseases mediated by this critical gene.

## 6 Conclusion

This study explored the impact of single nucleotide polymorphisms (SNPs) in the Bcl-2 gene on the protein structural and functional dynamics, with implications for carcinogenesis. Comprehensive bioinformatics tools and molecular dynamics simulations revealed that 8.5% of identified mutations in Bcl-2 were pathogenic, with Bcl-2^G101V^ and Bcl-2^F104L^ emerging as the most deleterious variants. These mutations significantly disrupted protein stability, binding affinities in protein-protein interactions, and ligand-binding capabilities. Structural and dynamic analyses indicated that these mutations led to conformational deviations, altered secondary structure, and compromised the integrity of critical functional motifs such as the BH3 domain.

The findings underscore the pivotal role of Bcl-2 mutations in disrupting apoptotic regulation, a hallmark of cancer, and highlight their potential as diagnostic biomarkers and therapeutic targets. By providing a detailed characterization of mutation-induced effects on Bcl-2, this study lays a foundation for future experimental validation and the development of targeted anti-cancer strategies, including rational drug design.

While the current work focuses on the molecular implications of Bcl-2 SNPs, future research integrating broader genomic and epigenetic datasets, as well as comparative analyses across species, will provide a more holistic understanding the role of Bcl-2 in apoptosis and oncogenesis. These efforts could contribute to innovative therapeutic interventions targeting Bcl-2-associated pathways in cancer.

## Data Availability

The original contributions presented in the study are included in the article/Supplementary Material, further inquiries can be directed to the corresponding author.

## References

[B65] Adams J. M. Cory S. (2017). The BCL-2 arbiters of apoptosis and their growing role as cancer targets. Cell Death Differ. 25 (1), 27–36. 10.1038/cdd.2017.161 29099483 PMC5729526

[B1] Adzhubei I. Jordan D. M. Sunyaev S. R. (2013). Predicting functional effect of human missense mutations using PolyPhen‐2. Curr. Protoc. Hum. Genet. 76 (1), 20. 10.1002/0471142905.hg0720s76 PMC4480630 23315928

[B2] Ashkenazy H. Abadi S. Martz E. Chay O. Mayrose I. Pupko T. (2016). ConSurf 2016: an improved methodology to estimate and visualize evolutionary conservation in macromolecules. Nucleic Acids Res. 44 (W1), W344–W350. 10.1093/nar/gkw408 27166375 PMC4987940

[B66] Barriuso D. Alvarez-Frutos L. Gonzalez-Gutierrez L. Motiño O. Kroemer G. Palacios-Ramirez R. (2023). Involvement of bcl-2 family proteins in tetraploidization-related senescence. Int. J. Mol. Sci. 24 (7), 6374. 10.3390/IJMS24076374 37047342 PMC10094710

[B3] Bateman A. Martin M. J. O’Donovan C. (2017). UniProt: the universal protein knowledgebase. Nucleic Acids Res. 45 (D1), D158–D169. 10.1093/NAR/GKW1099 27899622 PMC5210571

[B4] Bendl J. Stourac J. Salanda O. Pavelka A. Wieben E. D. Zendulka J. (2014). PredictSNP: robust and accurate consensus classifier for prediction of disease-related mutations. PLoS Comput. Biol. 10 (1), e1003440. 10.1371/journal.pcbi.1003440 24453961 PMC3894168

[B5] Berendsen H. J. C. Postma J. P. M. van Gunsteren W. F. DiNola A. Haak J. R. (1984). Molecular dynamics with coupling to an external bath. J. Chem. Phys. 81 (8), 3684–3690. 10.1063/1.448118

[B6] Birkinshaw R. W. Gong J. nan Luo C. S. Lio D. White C. A. Anderson M. A. (2019). Structures of BCL-2 in complex with venetoclax reveal the molecular basis of resistance mutations. Nat. Commun. 10 (1), 2385. 10.1038/s41467-019-10363-1 31160589 PMC6547681

[B7] Blombery P. Anderson M. A. Gong J. N. Thijssen R. Birkinshaw R. W. Thompson E. R. (2019). Acquisition of the recurrent Gly101Val mutation in BCL2 confers resistance to venetoclax in patients with progressive chronic lymphocytic leukemia. Cancer Discov. 9 (3), 342–353. 10.1158/2159-8290.CD-18-1119 30514704

[B8] Blombery P. Thompson E. R. Nguyen T. Birkinshaw R. W. Gong J. N. Chen X. (2020). Multiple BCL2 mutations cooccurring with Gly101Val emerge in chronic lymphocytic leukemia progression on venetoclax. Blood 135 (10), 773–777. 10.1182/BLOOD.2019004205 31951646 PMC7146015

[B9] Capriotti E. Calabrese R. Fariselli P. Martelli P. Altman R. B. Casadio R. (2013). WS-SNPs&GO: a web server for predicting the deleterious effect of human protein variants using functional annotation. BMC Genomics 14 (Suppl. 3), S6. 10.1186/1471-2164-14-S3-S6 PMC3665478 23819482

[B10] Capriotti E. Fariselli P. (2017). PhD-SNPg: a webserver and lightweight tool for scoring single nucleotide variants. Nucleic Acids Res. 45 (W1), W247–W252. 10.1093/NAR/GKX369 28482034 PMC5570245

[B11] Case D. A. Walker R. C. Cheatham T. E. Case D. A. Walker R. C. (2024). Amber 2018. Univ. Calif. San. Fr. 2018.

[B68] Chatterjee N. Walker G. C. (2017). Mechanisms of DNA damage, repair, and mutagenesis. Environ. Mol. Mutagen. 58 (5), 235–263. 10.1002/EM.22087 28485537 PMC5474181

[B12] Chen J. Zeng Q. Wang W. Sun H. Hu G. (2022). Decoding the identification mechanism of an SAM-III riboswitch on ligands through multiple independent Gaussian-accelerated molecular dynamics simulations. J. Chem. Inf. Model. 62 (23), 6118–6132. 10.1021/acs.jcim.2c00961 36440874

[B13] Chen J. Zhang S. Wang W. Pang L. Zhang Q. Liu X. (2021). Mutation-induced impacts on the switch transformations of the GDP- and GTP-bound K-ras: insights from multiple replica Gaussian accelerated molecular dynamics and free energy analysis. J. Chem. Inf. Model. 61 (4), 1954–1969. 10.1021/acs.jcim.0c01470 33739090

[B14] Chen Y. Lu H. Zhang N. Zhu Z. Wang S. Li M. (2020). PremPS: predicting the impact of missense mutations on protein stability. PLoS Comput. Biol. 16 (12), e1008543. 10.1371/journal.pcbi.1008543 33378330 PMC7802934

[B70] Conway P. J. Dao J. Kovalskyy D. Mahadevan D. Dray E. (2024). Polyploidy in cancer: causal mechanisms, cancer-specific consequences, and emerging treatments. Mol. Cancer Ther. 23 (5), 638–647. 10.1158/1535-7163 38315992 PMC11174144

[B15] Cory S. Adams J. M. (2002). The Bcl2 family: regulators of the cellular life-or-death switch. Nat. Rev. Cancer 2 (9), 647–656. 10.1038/nrc883 12209154

[B16] Czabotar P. E. Lessene G. Strasser A. Adams J. M. (2014). Control of apoptosis by the BCL-2 protein family: implications for physiology and therapy. Nat. Rev. Mol. Cell. Biol. 15 (1), 49–63. 10.1038/NRM3722 24355989

[B17] Dakal T. C. Kala D. Dhiman G. Yadav V. Krokhotin A. Dokholyan N. V. (2017). Predicting the functional consequences of non-synonymous single nucleotide polymorphisms in IL8 gene. Sci. Rep. 7 (1), 6525–6618. 10.1038/s41598-017-06575-4 28747718 PMC5529537

[B18] Darden T. York D. Pedersen L. (1993). Particle mesh Ewald: an N ⋅log(N) method for Ewald sums in large systems. J. Chem. Phys. 98 (12), 10089–10092. 10.1063/1.464397

[B19] Delbridge A. R. D. Strasser A. (2015). The BCL-2 protein family, BH3-mimetics and cancer therapy. Cell. Death Differ. 22 (7), 1071–1080. 10.1038/cdd.2015.50 25952548 PMC4572872

[B20] Delbridge ARDD Grabow S. Strasser A. Vaux D. L. (2016). Thirty years of BCL-2: translating cell death discoveries into novel cancer therapies. Nat. Rev. Cancer 16 (2), 99–109. 10.1038/nrc.2015.17 26822577

[B21] Edelman L. B. Eddy J. A. Price N. D. (2010). *In silico* models of cancer. WIREs Syst. Biol. Med. 2 (4), 438–459. 10.1002/wsbm.75 PMC3157287 20836040

[B22] Elamin G. Aljoundi A. E. S. Soliman M. (2024). From biological activity to stereoselectivity: a portrait of molecular and mechanistic profiles of the therapeutic potential of G-1 and LNS8801 as GPER-1 activator in the treatment of waldenström’s macroglobulinemia. Innov. Discov. 1, 7. 10.53964/id.2024007

[B23] Frappier V. Chartier M. Najmanovich R. J. (2015). ENCoM server: exploring protein conformational space and the effect of mutations on protein function and stability. Nucleic Acids Res. 43 (W1), W395–W400. 10.1093/nar/gkv343 25883149 PMC4489264

[B24] Goff D. J. Recart A. C. Sadarangani A. Chun H. J. Barrett C. L. Krajewska M. (2013). A pan-BCL2 inhibitor renders bone-marrow-resident human leukemia stem cells sensitive to tyrosine kinase inhibition. Cell. Stem Cell. 12 (3), 316–328. 10.1016/j.stem.2012.12.011 23333150 PMC3968867

[B25] Heinig M. Frishman D. (2004). STRIDE: a web server for secondary structure assignment from known atomic coordinates of proteins. Nucleic Acids Res. 32 (Web Server), W500–W502. 10.1093/nar/gkh429 15215436 PMC441567

[B26] Hubbard T. Barker D. Birney E. Cameron G. Chen Y. Clark L. (2002). The Ensembl genome database project. Nucleic Acids Res. 30 (1), 38–41. 10.1093/NAR/30.1.38 11752248 PMC99161

[B27] Humphrey W. Dalke A. Schulten K. (1996). VMD: Visual molecular dynamics. J. Mol. Graph 14 (1), 33–28. 10.1016/0263-7855(96)00018-5 8744570

[B67] Ichim G. Tait S. W. G. (2016). A fate worse than death: apoptosis as an oncogenic process. Nat. Rev. Cancer. 16 (8), 539–548. 10.1038/nrc.2016.58 27364482

[B28] Ioannidis N. M. Rothstein J. H. Pejaver V. Middha S. McDonnell S. K. Baheti S. (2016). REVEL: an Ensemble method for predicting the pathogenicity of Rare missense variants. Am. J. Hum. Genet. 99 (4), 877–885. 10.1016/j.ajhg.2016.08.016 27666373 PMC5065685

[B29] Jiménez‐Santos M. J. García‐Martín S. Fustero‐Torre C. Di Domenico T. Gómez‐López G. Al‐Shahrour F. (2022). Bioinformatics roadmap for therapy selection in cancer genomics. Mol. Oncol. 16 (21), 3881–3908. 10.1002/1878-0261.13286 35811332 PMC9627786

[B30] Jin J. Wu X. Yin J. Li M. Shen J. Li J. (2019). Identification of genetic mutations in cancer: challenge and opportunity in the new era of targeted therapy. Front. Oncol. 9, 263. 10.3389/fonc.2019.00263 31058077 PMC6477148

[B69] Kalkavan H. Rühl S. Shaw J. J. P. Green D. R. (2023). Non-lethal outcomes of engaging regulated cell death pathways in cancer. Nat. Cancer. 4 (6), 795–806. 10.1038/s43018-023-00571-6 37277528 PMC10416134

[B31] Karimi M. R. Karimi A. H. Abolmaali S. Sadeghi M. Schmitz U. (2022). Prospects and challenges of cancer systems medicine: from genes to disease networks. Brief. Bioinform 23 (1), bbab343. 10.1093/bib/bbab343 34471925 PMC8769701

[B32] Kitada S. Pedersen I. M. Schimmer A. D. Reed J. C. (2002). Dysregulation of apoptosis genes in hematopoietic malignancies. Oncogene 21 (21), 3459–3474. 10.1038/sj.onc.1205327 12032782

[B33] Kucukkal T. G. Petukh M. Li L. Alexov E. (2015). Structural and physico-chemical effects of disease and non-disease nsSNPs on proteins. Curr. Opin. Struct. Biol. 32, 18–24. 10.1016/j.sbi.2015.01.003 25658850 PMC4511717

[B34] Kumalo H. M. Bhakat S. Soliman M. E. (2016). Investigation of flap flexibility of β-secretase using molecular dynamic simulations. J. Biomol. Struct. Dyn. 34 (5), 1008–1019. 10.1080/07391102.2015.1064831 26208540

[B35] Kumar P. Henikoff S. Ng P. C. (2009). Predicting the effects of coding non-synonymous variants on protein function using the SIFT algorithm. Nat. Protoc. 4 (7), 1073–1081. 10.1038/NPROT.2009.86 19561590

[B71] Lavrik I. Golks A. Krammer P. H. (2005). Death receptor signaling. J. Cell Sci. 118 (2), 265–267. 10.1242/JCS.01610 15654015

[B36] Liu X. Wu C. Li C. Boerwinkle E. (2016). dbNSFP v3.0: a one-stop database of functional predictions and annotations for human nonsynonymous and splice-site SNVs. Hum. Mutat. 37 (3), 235–241. 10.1002/humu.22932 26555599 PMC4752381

[B37] López-Ferrando V. Gazzo A. de la Cruz X. Orozco M. Gelpí J. L. (2017). PMut: a web-based tool for the annotation of pathological variants on proteins, 2017 update. Nucleic Acids Res. 45 (W1), W222–W228. 10.1093/nar/gkx313 28453649 PMC5793831

[B38] Maier J. A. Martinez C. Kasavajhala K. Wickstrom L. Hauser K. E. Simmerling C. (2015). ff14SB: improving the accuracy of protein side chain and backbone parameters from ff99SB. J. Chem. Theory Comput. 11 (8), 3696–3713. 10.1021/acs.jctc.5b00255 26574453 PMC4821407

[B73] McDonald P. C. Nagel J. M. Dedhar S. (2021). Anastasis, recovery from the brink of death as a mechanism of drug resistance. Biological Mechanisms and the Advancing Approaches to Overcoming Cancer Drug Resistance. 251–260. 10.1016/B978-0-12-821310-0.00004-8

[B72] Nano M. Montell D. J. (2024). Apoptotic signaling: beyond cell death. Semin. Cell Dev. Biol. 156, 22–34. 10.1016/J.SEMCDB.2023.11.002 37988794

[B39] Ngan C. H. Hall D. R. Zerbe B. Grove L. E. Kozakov D. Vajda S. (2012). FTSite: high accuracy detection of ligand binding sites on unbound protein structures. Bioinformatics 28 (2), 286–287. 10.1093/bioinformatics/btr651 22113084 PMC3259439

[B76] Niculescu V. (2024). Re-Evaluating cancer stem cells (CSCs) and polyploid giant cancer cells (PGCCs) in the light of evolutionary cancer cell biology ECCB. [Epub ahead of print]. 10.20944/PREPRINTS202409.0026.V1

[B74] Niculescu V. F. (2024). Understanding cancer from an evolutionary perspective: high-risk reprogramming of genome-damaged stem cells. Acad. Med. [Epub ahead of print]. 10.20935/ACADMED6168

[B75] Niculescu V. F. Niculescu E. R. (2024). The enigma of cancer polyploidy as deciphered by evolutionary cancer stem cell biology (ECCB). Acad. Med. [Epub ahead of print]. 10.20935/ACADMED6233

[B40] Parthiban V. Gromiha M. M. Schomburg D. (2006). CUPSAT: prediction of protein stability upon point mutations. Nucleic Acids Res. 34 (Web Server), W239–W242. 10.1093/nar/gkl190 16845001 PMC1538884

[B41] Pejaver V. Urresti J. Lugo-Martinez J. Pagel K. A. Lin G. N. Nam H. J. (2020). Inferring the molecular and phenotypic impact of amino acid variants with MutPred2. Nat. Commun. 11 (1), 5918–6013. 10.1038/s41467-020-19669-x 33219223 PMC7680112

[B42] Perini G. F. Ribeiro G. N. Pinto Neto J. V. Campos L. T. Hamerschlak N. (2018). BCL-2 as therapeutic target for hematological malignancies. J. Hematol. Oncol. 11 (1), 65. 10.1186/s13045-018-0608-2 29747654 PMC5946445

[B43] Pires D. E. V. Ascher D. B. Blundell T. L. (2014a). mCSM: predicting the effects of mutations in proteins using graph-based signatures. Bioinformatics 30 (3), 335–342. 10.1093/bioinformatics/btt691 24281696 PMC3904523

[B44] Pires D. E. V. Ascher D. B. Blundell T. L. (2014b). DUET: a server for predicting effects of mutations on protein stability using an integrated computational approach. Nucleic Acids Res. 42 (W1), W314–W319. 10.1093/nar/gku411 24829462 PMC4086143

[B45] Qian S. Wei Z. Yang W. Huang J. Yang Y. Wang J. (2022). The role of BCL-2 family proteins in regulating apoptosis and cancer therapy. Front. Oncol. 12, 985363. 10.3389/fonc.2022.985363 36313628 PMC9597512

[B46] Rentzsch P. Witten D. Cooper G. M. Shendure J. Kircher M. (2019). CADD: predicting the deleteriousness of variants throughout the human genome. Nucleic Acids Res. 47 (D1), D886–D894. 10.1093/nar/gky1016 30371827 PMC6323892

[B47] Reva B. Antipin Y. Sander C. (2011). Predicting the functional impact of protein mutations: application to cancer genomics. Nucleic Acids Res. 39 (17), e118. 10.1093/nar/gkr407 21727090 PMC3177186

[B48] Roberts A. W. Davids M. S. Pagel J. M. Kahl B. S. Puvvada S. D. Gerecitano J. F. (2016). Targeting BCL2 with venetoclax in relapsed chronic lymphocytic leukemia. N. Engl. J. Med. 374 (4), 311–322. 10.1056/NEJMoa1513257 26639348 PMC7107002

[B49] Rodrigues C. H. Pires D. E. Ascher D. B. (2018). DynaMut: predicting the impact of mutations on protein conformation, flexibility and stability. Nucleic Acids Res. 46 (W1), W350–W355. 10.1093/nar/gky300 29718330 PMC6031064

[B50] Rodrigues C. H. M. Myung Y. Pires D. E. V. Ascher D. B. (2019). mCSM-PPI2: predicting the effects of mutations on protein–protein interactions. Nucleic Acids Res. 47 (W1), W338–W344. 10.1093/nar/gkz383 31114883 PMC6602427

[B51] Roe D. R. Cheatham T. E. (2013). PTRAJ and CPPTRAJ: software for processing and analysis of molecular dynamics trajectory data. J. Chem. Theory Comput. 9 (7), 3084–3095. 10.1021/ct400341p 26583988

[B52] Rosser C. J. Reyes A. O. Vakar-Lopez F. Levy L. B. Kuban D. A. Hoover D. C. (2003). Bcl-2 is significantly overexpressed in localized radio-recurrent prostate carcinoma, compared with localized radio-naive prostate carcinoma. Int. J. Radiat. Oncology*Biology*Physics 56 (1), 1–6. 10.1016/S0360-3016(02)04468-1 12694817

[B53] Seifert E. (2014). OriginPro 9.1: scientific data analysis and graphing software-software review. J. Chem. Inf. Model. 54 (5), 1552. 10.1021/ci500161d 24702057

[B54] Sherry S. T. Ward M. H. Kholodov M. Baker J. Phan L. Smigielski E. M. (2001). dbSNP: the NCBI database of genetic variation. Nucleic Acids Res. 29 (1), 308–311. 10.1093/NAR/29.1.308 11125122 PMC29783

[B55] Shivakumar D. Harder E. Damm W. Friesner R. A. Sherman W. (2012). Improving the prediction of absolute solvation free energies using the next generation OPLS force field. J. Chem. Theory Comput. 8 (8), 2553–2558. 10.1021/ct300203w 26592101

[B56] Stilgenbauer S. Eichhorst B. Schetelig J. Hillmen P. Seymour J. F. Coutre S. (2018). Venetoclax for patients with chronic lymphocytic leukemia with 17p deletion: results from the full population of a phase II pivotal trial. J. Clin. Oncol. 36 (19), 1973–1980. 10.1200/JCO.2017.76.6840 29715056

[B57] Szklarczyk D. Gable A. L. Nastou K. C. Lyon D. Kirsch R. Pyysalo S. (2021). The STRING database in 2021: customizable protein–protein networks, and functional characterization of user-uploaded gene/measurement sets. Nucleic Acids Res. 49 (D1), D605–D612. 10.1093/nar/gkaa1074 33237311 PMC7779004

[B58] Tang X. Wuest M. Benesch M. G. K. Dufour J. Zhao Y. Curtis J. M. (2020). Inhibition of autotaxin with GLPG1690 increases the efficacy of radiotherapy and chemotherapy in a mouse model of breast cancer. Mol. Cancer Ther. 19 (1), 63–74. 10.1158/1535-7163.MCT-19-0386 31548293

[B59] Thomas P. D. Ebert D. Muruganujan A. Mushayahama T. Albou L. P. Mi H. (2022). PANTHER: making genome-scale phylogenetics accessible to all. Protein Sci. 31 (1), 8–22. 10.1002/pro.4218 34717010 PMC8740835

[B77] Vasileva M. H. Bennemann A. Zachmann K. Schön M. P. Frank J. Ulaganathan V. K. (2024). CD24 flags anastasis in melanoma cells. Apoptosis, 1–15. 10.1007/S10495-024-01990-1/FIGURES/12 39136818 PMC11799124

[B60] Wang J. Q. Li J. Y. Teng Q. X. Lei Z. N. Ji N. Cui Q. (2020). Venetoclax, a BCL-2 inhibitor, enhances the efficacy of chemotherapeutic agents in wild-type ABCG2-overexpression-mediated MDR cancer cells. Cancers (Basel) 12 (2), 466. 10.3390/CANCERS12020466 32085398 PMC7072352

[B61] Warde-Farley D. Donaldson S. L. Comes O. Zuberi K. Badrawi R. Chao P. (2010). The GeneMANIA prediction server: biological network integration for gene prioritization and predicting gene function. Nucleic Acids Res. 38, W214–W220. 10.1093/NAR/GKQ537 20576703 PMC2896186

[B62] Xue Y. Zhou F. Zhu M. Ahmed K. Chen G. Yao X. (2005). GPS: a comprehensive www server for phosphorylation sites prediction. Nucleic Acids Res. 33 (Web Server), W184–W187. 10.1093/nar/gki393 15980451 PMC1160154

[B63] Yalcin-Ozkat G. (2021). Molecular modeling strategies of cancer multidrug resistance. Drug Resist. Updat. 59, 100789. 10.1016/j.drup.2021.100789 34973929

[B64] Yin X. M. Oltvai Z. N. Korsmeyer S. J. (1994). BH1 and BH2 domains of Bcl-2 are required for inhibition of apoptosis and heterodimerization with Bax. Nature 369 (6478), 321–323. 10.1038/369321a0 8183370

[B78] Youle R. J. Strasser A. (2008). The BCL-2 protein family: opposing activities that mediate cell death. Nat. Rev. Mol. Cell Biol. 9 (1), 47–59. 10.1038/nrm2308 18097445

[B79] Zaitceva V. Kopeina G. S. Zhivotovsky B. (2021). Anastasis: return journey from cell death. Cancers. 13 (15), 3671. 10.3390/CANCERS13153671 34359573 PMC8345212

