# Erratum: Integrative genomic analyses combined with molecular dynamics simulations reveal the impact of deleterious mutations of Bcl-2 gene on the apoptotic machinery and implications in carcinogenesis

**DOI:** 10.3389/fgene.2025.1558506

**Published:** 2025-01-23

**Authors:** Frontiers Production Office

**Affiliations:** Frontiers Media SA, Lausanne, Switzerland

**Keywords:** Bcl-2, nsSNPs, mutations, genomic analyses, molecular dynamics simulations

Due to a production error, the latest version of the article was not published.

Corrections have been made to the Abstract, Introduction, Discussion, and Conclusion sections. Furthermore, a new section called Limitations of the Study and Future Perspectives has been added.

The publisher apologizes for this mistake. The original version of this article has been updated.

