# Supplementary material for: Integrative genomic analyses combined with molecular dynamics simulations reveal the impact of deleterious mutations of Bcl-2 gene on the apoptotic machinery and implications in carcinogenesis
Source: Front Genet. 2025 Jan 7;15:1502152. doi: 10.3389/fgene.2024.1502152 (PMC11747654; doi:10.3389/fgene.2024.1502152)
Supplement: Supplementary file 1 [file Table1.docx]

**Integrative Genomic Analyses Combined with Molecular Dynamics Simulations Reveal the Impact of Deleterious Mutations of Bcl-2 Gene on the Apoptotic Machinery and Implications in Carcinogenesis**

**Ghazi Elamin^1^’^2^, Zhichao Zhang^3^, Depika Dwarka^4^, Kabange Kasumbwe^5^, John Mellem^5^ Nompumelelo P. Mkhwanazi^6^, Paradise Madlala^6,7^ and Mahmoud E.S Soliman^1*^**

^1^ Molecular Bio-computation and Drug Design Laboratory, School of Health Sciences, University of KwaZulu-Natal, Westville Campus, Durban 4001, South Africa ([soliman@ukzn.ac.za](mailto:soliman@ukzn.ac.za)) ([ballag1@ukzn.ac.za](mailto:ballag1@ukzn.ac.za))

^2^ Department of Pharmaceutical Chemistry, College of Pharmacy, Karary University, Khartoum P.O. Box 11111, Sudan

^3^ School of Chemistry, Dalian University of Technology, Dalian, Liaoning 116024, China ([zczhang@dlut.edu.cn](mailto:zczhang@dlut.edu.cn))

^4^Ezintsha, Faculty of Health Sciences, University of Witwatersrand, Johannesburg, South Africa ([DDwarka@ezintsha.org](mailto:DDwarka@ezintsha.org))

^5^Biotechnology and Food Science, Durban University of Technology, Durban, South Africa ([johnm@dut.ac.za](mailto:johnm@dut.ac.za))

^6^HIV Pathogenesis Programme, School of Laboratory Medicine and Medical Science, The Doris Duke Medical Research Institute, Nelson R. Mandela School of Medicine, University of KwaZulu-Natal, Durban, South Africa ([mkhwanazi@ukzn.ac.za](mailto:mkhwanazi@ukzn.ac.za))

^7^ School of Laboratory Medicine and Medical Sciences, University of KwaZulu-Natal, Durban, South Africa ([madlalap@ukzn.ac.za](mailto:madlalap@ukzn.ac.za))

*Correspondence: [soliman@ukzn.ac.za](mailto:soliman@ukzn.ac.za): (<http://soliman.ukzn.ac.za>

**Table S1.** Sequence-based prediction of mutations associated with Bcl-2.

| **S. No.** | **Variant ID** | **Variant** | **Predictor** | | | | | | | |
| --- | --- | --- | --- | --- | --- | --- | --- | --- | --- | --- |
|  |  |  | **SIFT** | **PolyPhen2** | **CADD** | **REVEL** | **MetaLR** | **Mutation assessor** | **FATHMM** | **predict-SNP** |
| 1. | rs775404824 | G8E | Deleterious | Deleterious | Tolerated | Tolerated | Tolerated | Deleterious | Tolerated | Deleterious |
| 2. | [rs769588208](https://www.ensembl.org/Homo_sapiens/Variation/Explore?db=core;g=ENSG00000171791;r=18:63123346-63319769;t=ENST00000333681;vf=77768199) | N11S | Tolerated | Deleterious | Tolerated | Tolerated | Tolerated | Deleterious | Tolerated | Deleterious |
| 3. | rs960653284 | R12G | Deleterious | Deleterious | Tolerated | Tolerated | Tolerated | Deleterious | Deleterious | Deleterious |
| 4. | rs745851862 | V15L | Deleterious | Deleterious | Tolerated | Tolerated | Tolerated | Deleterious | Deleterious | Deleterious |
| 5. | rs776360417 | M16I | Tolerated | Tolerated | Tolerated | Tolerated | Tolerated | Tolerated | Tolerated | Tolerated |
| 6. | rs565741123 | K17N | Tolerated | Tolerated | Tolerated | Tolerated | Tolerated | Tolerated | Deleterious | Tolerated |
| 7. | rs780634396 | K17E | Deleterious | Deleterious | Tolerated | Tolerated | Tolerated | Deleterious | Deleterious | Tolerated |
| 8. | rs746600883 | I19L | Tolerated | Tolerated | Tolerated | Tolerated | Tolerated | Tolerated | Tolerated | Tolerated |
| 9. | rs758123306 | H20Q | Tolerated | Deleterious | Tolerated | Tolerated | Tolerated | Deleterious | Tolerated | Tolerated |
| 10. | rs777401949 | H20N | Tolerated | Possibly damaging | Tolerated | Tolerated | Tolerated | Tolerated | Tolerated | Tolerated |
| 11. | rs1441603213 | L23M | Deleterious | Deleterious | Tolerated | Tolerated | Tolerated | Deleterious | Deleterious | Deleterious |
| 12. | rs779372254 | G27S | Deleterious | Deleterious | Tolerated | Tolerated | Tolerated | Deleterious | Deleterious | Deleterious |
| 13. | rs1555711318 | Y28H | Tolerated | Deleterious | Tolerated | Tolerated | Tolerated | Deleterious | Deleterious | Deleterious |
| 14. | rs766508625 | D31H | Deleterious | Possibly damaging | Tolerated | Tolerated | Tolerated | Tolerated | Tolerated | Tolerated |
| 15. | rs546449806 | A32G | Tolerated | Tolerated | Tolerated | Tolerated | Tolerated | Tolerated | Tolerated | Tolerated |
| 16. | rs760840889 | A32P | Tolerated | Possibly damaging | Tolerated | Tolerated | Tolerated | Tolerated | Tolerated | Tolerated |
| 17. | rs767612613 | G33R | Tolerated | Deleterious | Tolerated | Tolerated | Tolerated | Deleterious | Tolerated | Tolerated |
| 18. | rs540701354 | D34Y | Deleterious | Possibly damaging | Tolerated | Tolerated | Tolerated | Tolerated | Deleterious | Deleterious |
| 19. | rs775108608 | V35M | Tolerated | Tolerated | Tolerated | Tolerated | Tolerated | Tolerated | Tolerated | Tolerated |
| 20. | rs1224885133 | P39L | Tolerated | Tolerated | Tolerated | Tolerated | Tolerated | Tolerated | Tolerated | Tolerated |
| 21. | rs770925898 | P40L | Tolerated | Tolerated | Tolerated | Tolerated | Tolerated | Tolerated | Tolerated | Tolerated |
| 22. | rs777309369 | G41R | Tolerated | Tolerated | Tolerated | Tolerated | Tolerated | Tolerated | Tolerated | Tolerated |
| 23. | rs1235481489 | A42T | Tolerated | Tolerated | Tolerated | Tolerated | Tolerated | Tolerated | Tolerated | Tolerated |
| 24. | rs1800477 | A43T | Tolerated | Possibly damaging | Tolerated | Tolerated | Tolerated | Tolerated | Tolerated | Tolerated |
| 25. | rs779096658 | A45S | Tolerated | Tolerated | Tolerated | Tolerated | Tolerated | Tolerated | Tolerated | Tolerated |
| 26. | rs755382644 | A45G | Tolerated | Tolerated | Tolerated | Tolerated | Tolerated | Tolerated | Tolerated | Tolerated |
| 27. | rs749745319 | P46A | Tolerated | Tolerated | Tolerated | Tolerated | Tolerated | Tolerated | Tolerated | Tolerated |
| 28. | rs369277721 | P46L | Tolerated | Tolerated | Tolerated | Tolerated | Tolerated | Tolerated | Tolerated | Tolerated |
| 29. | rs1448547701 | G47V | Tolerated | Tolerated | Tolerated | Tolerated | Tolerated | Tolerated | Tolerated | Tolerated |
| 30. | rs368344129 | G47S | Tolerated | Tolerated | Tolerated | Tolerated | Tolerated | Tolerated | Tolerated | Tolerated |
| 31. | rs1351068431 | S51P | Tolerated | Tolerated | Tolerated | Tolerated | Tolerated | Tolerated | Tolerated | Tolerated |
| 32. | rs1324162631 | Q52S | Tolerated | Tolerated | Tolerated | Tolerated | Tolerated | Tolerated | Tolerated | Tolerated |
| 33. | rs1245727781 | G54R | Tolerated | Tolerated | Tolerated | Tolerated | Tolerated | Tolerated | Tolerated | Tolerated |
| 34. | rs776473075 | H55Q | Tolerated | Tolerated | Tolerated | Tolerated | Tolerated | Tolerated | Tolerated | Tolerated |
| 35. | rs759325300 | H55Y | Deleterious | Tolerated | Tolerated | Tolerated | Tolerated | Tolerated | Tolerated | Tolerated |
| 36. | rs766277782 | T56R | Tolerated | Tolerated | Tolerated | Tolerated | Tolerated | Tolerated | Tolerated | Tolerated |
| 37. | rs1192476761 | P59Q | Tolerated | Tolerated | Tolerated | Tolerated | Tolerated | Tolerated | Tolerated | Tolerated |
| 38. | rs1409089747 | A60V | Tolerated | Tolerated | Tolerated | Tolerated | Tolerated | Tolerated | Tolerated | Tolerated |
| 39. | rs772554403 | A61S | Tolerated | Tolerated | Tolerated | Tolerated | Tolerated | Tolerated | Tolerated | Tolerated |
| 40. | rs747696230 | S62F | Deleterious | Tolerated | Tolerated | Tolerated | Tolerated | Tolerated | Deleterious | Deleterious |
| 41. | rs1450331336 | R63P | Tolerated | Tolerated | Tolerated | Tolerated | Tolerated | Tolerated | Tolerated | Tolerated |
| 42. | rs774056251 | R63W | Deleterious | Possibly damaging | Tolerated | Tolerated | Tolerated | Tolerated | Deleterious | Tolerated |
| 43. | rs1241412160 | P65L | Tolerated | Tolerated | Tolerated | Tolerated | Tolerated | Tolerated | Tolerated | Tolerated |
| 44. | rs768259110 | P65S | Tolerated | Tolerated | Tolerated | Tolerated | Tolerated | Tolerated | Tolerated | Tolerated |
| 45. | rs749649089 | V66I | Tolerated | Tolerated | Tolerated | Tolerated | Tolerated | Tolerated | Tolerated | Tolerated |
| 46. | rs756650851 | A67V | Tolerated | Tolerated | Tolerated | Tolerated | Tolerated | Tolerated | Tolerated | Tolerated |
| 47. | rs780606244 | A67S | Tolerated | Tolerated | Tolerated | Tolerated | Tolerated | Tolerated | Tolerated | Tolerated |
| 48. | rs746401408 | R68G | Tolerated | Tolerated | Tolerated | Tolerated | Tolerated | Tolerated | Tolerated | Tolerated |
| 49. | rs1338401488 | T69S | Tolerated | Tolerated | Tolerated | Tolerated | Tolerated | Tolerated | Tolerated | Tolerated |
| 50. | rs1435116050 | S70L | Tolerated | Tolerated | Tolerated | Tolerated | Tolerated | Tolerated | Tolerated | Tolerated |
| 51. | rs1486963186 | P71L | Deleterious | Possibly damaging | Tolerated | Tolerated | Tolerated | Tolerated | Tolerated | Deleterious |
| 52. | rs1304844332 | P71S | Tolerated | Possibly damaging | Tolerated | Tolerated | Tolerated | Tolerated | Tolerated | Tolerated |
| 53. | rs1299930433 | T74I | Tolerated | Tolerated | Tolerated | Tolerated | Tolerated | Tolerated | Tolerated | Tolerated |
| 54. | rs1599310991 | T74P | Tolerated | Tolerated | Tolerated | Tolerated | Tolerated | Tolerated | Tolerated | Tolerated |
| 55. | rs757360142 | P75L | Tolerated | Tolerated | Tolerated | Tolerated | Tolerated | Tolerated | Tolerated | Tolerated |
| 56. | rs1228330940 | A76T | Tolerated | Tolerated | Tolerated | Tolerated | Tolerated | Tolerated | Tolerated | Tolerated |
| 57. | rs1157110679 | A77V | Tolerated | Tolerated | Tolerated | Tolerated | Tolerated | Tolerated | Tolerated | Tolerated |
| 58. | rs753603356 | P78S | Tolerated | Tolerated | Tolerated | Tolerated | Tolerated | Tolerated | Tolerated | Tolerated |
| 59. | rs766196038 | G79R | Tolerated | Tolerated | Tolerated | Tolerated | Tolerated | Tolerated | Tolerated | Tolerated |
| 60. | rs760559670 | A80V | Tolerated | Tolerated | Tolerated | Tolerated | Tolerated | Tolerated | Tolerated | Tolerated |
| 61. | rs1477078177 | A80S | Tolerated | Tolerated | Tolerated | Tolerated | Tolerated | Tolerated | Tolerated | Tolerated |
| 62. | rs201827119 | A82V | Tolerated | Tolerated | Tolerated | Tolerated | Tolerated | Tolerated | Tolerated | Tolerated |
| 63. | rs375594294 | G83E | Tolerated | Tolerated | Tolerated | Tolerated | Tolerated | Tolerated | Tolerated | Tolerated |
| 64. | rs761382113 | P84S | Tolerated | Tolerated | Tolerated | Tolerated | Tolerated | Tolerated | Tolerated | Tolerated |
| 65. | rs770293857 | S87R | Tolerated | Tolerated | Tolerated | Tolerated | Tolerated | Tolerated | Tolerated | Tolerated |
| 66. | rs748978916 | S87G | Tolerated | Tolerated | Tolerated | Tolerated | Tolerated | Tolerated | Tolerated | Tolerated |
| 67. | rs781718615 | P88L | Tolerated | Deleterious | Tolerated | Tolerated | Tolerated | Tolerated | Tolerated | Tolerated |
| 68. | rs1467376730 | V89A | Tolerated | Tolerated | Tolerated | Tolerated | Tolerated | Tolerated | Tolerated | Tolerated |
| 69. | rs1467376730 | V93A | Deleterious | Possibly damaging | Tolerated | Tolerated | Tolerated | Tolerated | Tolerated | Deleterious |
| 70. | rs551395951 | H94P | Deleterious | Deleterious | Tolerated | Tolerated | Tolerated | Tolerated | Tolerated | Deleterious |
| 71. | rs373889054 | L95V | Tolerated | Tolerated | Tolerated | Tolerated | Tolerated | Tolerated | Tolerated | Tolerated |
| 72. | rs752942153 | T96A | Tolerated | Tolerated | Tolerated | Tolerated | Tolerated | Tolerated | Tolerated | Tolerated |
| 73. | rs528042823 | L97P | Deleterious | Deleterious | Deleterious | Deleterious | Tolerated | Deleterious | Deleterious | Deleterious |
| 74. | rs755908754 | R98L | Deleterious | Deleterious | Tolerated | Tolerated | Tolerated | Deleterious | Tolerated | Deleterious |
| 75. | rs559397524 | Q99R | Tolerated | Tolerated | Tolerated | Tolerated | Tolerated | Tolerated | Tolerated | Tolerated |
| 76. | rs750253286 | A100V | Deleterious | Deleterious | Deleterious | Tolerated | Tolerated | Tolerated | Tolerated | Tolerated |
| 77. | rs1184034130 | A100T | Deleterious | Deleterious | Tolerated | Tolerated | Tolerated | Deleterious | Tolerated | Tolerated |
| 78. | rs1208924867 | G101V | Deleterious | Deleterious | Deleterious | Deleterious | Tolerated | Deleterious | Deleterious | Deleterious |
| 79. | rs761696930 | D102N | Deleterious | Deleterious | Deleterious | Tolerated | Tolerated | Deleterious | Deleterious | Tolerated |
| 80. | rs1259350145 | D103Y | Deleterious | Deleterious | Deleterious | Tolerated | Tolerated | Tolerated | Deleterious | Deleterious |
| 81. | rs751038951 | F104L | Deleterious | Deleterious | Deleterious | Tolerated | Tolerated | Deleterious | Tolerated | Tolerated |
| 82. | rs762635201 | S105F | Deleterious | Deleterious | Deleterious | Tolerated | Tolerated | Tolerated | Deleterious | Deleterious |
| 83. | rs763718170 | S105P | Deleterious | Deleterious | Tolerated | Tolerated | Tolerated | Tolerated | Tolerated | Deleterious |
| 84. | rs1302520231 | R109H | Tolerated | Possibly damaging | Tolerated | Tolerated | Tolerated | Tolerated | Tolerated | Tolerated |
| 85. | rs1379595021 | R109C | Deleterious | Deleterious | Deleterious | Tolerated | Tolerated | Tolerated | Deleterious | Deleterious |
| 86. | rs990629198 | A113D | Tolerated | Tolerated | Tolerated | Tolerated | Tolerated | Tolerated | Tolerated | Tolerated |
| 87. | rs1284045765 | M115I | Tolerated | Tolerated | Tolerated | Tolerated | Tolerated | Tolerated | Tolerated | Tolerated |
| 88. | rs759928495 | Q118R | Tolerated | Deleterious | Tolerated | Tolerated | Tolerated | Deleterious | Tolerated | Deleterious |
| 89. | rs776905089 | L119M | Tolerated | Deleterious | Tolerated | Tolerated | Tolerated | Deleterious | Deleterious | Deleterious |
| 90. | rs373065228 | T122M | Deleterious | Deleterious | Deleterious | Tolerated | Tolerated | Deleterious | Deleterious | Deleterious |
| 91. | rs61733415 | F124L | Tolerated | Tolerated | Tolerated | Tolerated | Tolerated | Tolerated | Tolerated | Tolerated |
| 92. | rs1255855616 | R127Q | Tolerated | Tolerated | Tolerated | Tolerated | Tolerated | Tolerated | Tolerated | Tolerated |
| 93. | rs747504890 | G128R | Tolerated | Tolerated | Tolerated | Tolerated | Tolerated | Tolerated | Tolerated | Tolerated |
| 94. | rs777784952 | R129S | Tolerated | Possibly damaging | Tolerated | Tolerated | Tolerated | Tolerated | Tolerated | Tolerated |
| 95. | rs1239600576 | R129P | Deleterious | Deleterious | Deleterious | Tolerated | Tolerated | Deleterious | Tolerated | Deleterious |
| 96. | rs777784952 | R129C | Deleterious | Deleterious | Deleterious | Tolerated | Tolerated | Tolerated | Tolerated | Deleterious |
| 97. | rs772156175 | A131V | Tolerated | Tolerated | Tolerated | Tolerated | Tolerated | Tolerated | Tolerated | Tolerated |
| 98. | rs1368843367 | V134L | Deleterious | Possibly damaging | Tolerated | Tolerated | Tolerated | Tolerated | Tolerated | Tolerated |
| 99. | rs1005748945 | D140G | Deleterious | Deleterious | Deleterious | Deleterious | Tolerated | Deleterious | Tolerated | Deleterious |
| 100. | rs1473616979 | D140N | Deleterious | Deleterious | Deleterious | Tolerated | Tolerated | Deleterious | Tolerated | Deleterious |
| 101. | rs755252289 | G141E | Deleterious | Deleterious | Deleterious | Tolerated | Tolerated | Deleterious | Deleterious | Deleterious |
| 102. | rs1599310706 | V142G | Deleterious | Deleterious | Deleterious | Deleterious | Tolerated | Deleterious | Deleterious | Deleterious |
| 103. | rs1388498866 | N143S | Deleterious | Deleterious | Deleterious | Tolerated | Tolerated | Deleterious | Deleterious | Deleterious |
| 104. | rs780880487 | F150L | Deleterious | Deleterious | Deleterious | Tolerated | Tolerated | Tolerated | Tolerated | Tolerated |
| 105. | rs1446199866 | V156F | Deleterious | Deleterious | Tolerated | Tolerated | Tolerated | Tolerated | Tolerated | Deleterious |
| 106. | rs942677296 | M157L | Tolerated | Tolerated | Tolerated | Tolerated | Tolerated | Tolerated | Tolerated | Tolerated |
| 107. | rs1334020924 | V162F | Deleterious | Deleterious | Tolerated | Tolerated | Tolerated | Deleterious | Deleterious | Deleterious |
| 108. | rs1295831156 | M166I | Deleterious | Deleterious | Tolerated | Tolerated | Tolerated | Deleterious | Tolerated | Deleterious |
| 109. | rs201085318 | M166T | Deleterious | Deleterious | Tolerated | Deleterious | Tolerated | Deleterious | Tolerated | Deleterious |
| 110. | rs1219454995 | M166L | Deleterious | Deleterious | Tolerated | Tolerated | Tolerated | Tolerated | Tolerated | Deleterious |
| 111. | rs751464195 | P168S | Tolerated | Tolerated | Tolerated | Tolerated | Tolerated | Tolerated | Tolerated | Tolerated |
| 112. | rs1441924243 | N172S | Tolerated | Tolerated | Tolerated | Tolerated | Tolerated | Tolerated | Tolerated | Tolerated |
| 113. | rs909849040 | N172H | Tolerated | Deleterious | Tolerated | Tolerated | Tolerated | Tolerated | Tolerated | Tolerated |
| 114. | rs1228672103 | I173V | Tolerated | Deleterious | Tolerated | Tolerated | Tolerated | Tolerated | Tolerated | Tolerated |
| 115. | rs1178840163 | A174T | Deleterious | Deleterious | Tolerated | Tolerated | Tolerated | Tolerated | Tolerated | Tolerated |
| 116. | rs1599310597 | E179D | Tolerated | Tolerated | Tolerated | Tolerated | Tolerated | Tolerated | Tolerated | Tolerated |
| 117. | rs752310933 | L181V | Tolerated | Deleterious | Tolerated | Tolerated | Tolerated | Deleterious | Deleterious | Deleterious |
| 118. | rs1599310574 | R183Q | Tolerated | Tolerated | Tolerated | Tolerated | Tolerated | Tolerated | Tolerated | Tolerated |
| 119. | rs868435115 | R183W | Deleterious | Deleterious | Deleterious | Tolerated | Tolerated | Tolerated | Deleterious | Deleterious |
| 120. | rs1310296388 | H186R | Tolerated | Tolerated | Tolerated | Tolerated | Tolerated | Tolerated | Tolerated | Tolerated |
| 121. | rs1197820694 | G193R | Deleterious | Deleterious | Deleterious | Deleterious | Tolerated | Deleterious | Deleterious | Deleterious |
| 122. | rs1261263162 | A197D | Deleterious | Tolerated | Tolerated | Tolerated | Tolerated | Tolerated | Tolerated | Tolerated |
| 123. | rs1344438049 | A197T | Tolerated | Tolerated | Tolerated | Tolerated | Tolerated | Tolerated | Tolerated | Tolerated |
| 124. | rs1319293137 | E200D | Tolerated | Tolerated | Tolerated | Tolerated | Tolerated | Tolerated | Tolerated | Tolerated |
| 125. | rs148811059 | G203S | Tolerated | Tolerated | Tolerated | Tolerated | Tolerated | Tolerated | Tolerated | Tolerated |
| 126. | rs1380222499 | P204L | Tolerated | Tolerated | Tolerated | Tolerated | Tolerated | Tolerated | Tolerated | Tolerated |
| 127. | rs1335816980 | S205G | Tolerated | Tolerated | Tolerated | Tolerated | Tolerated | Tolerated | Tolerated | Tolerated |
| 128. | rs758367430 | M206V | Tolerated | Tolerated | Tolerated | Tolerated | Tolerated | Tolerated | Tolerated | Tolerated |
| 129. | rs369294037 | R207Q | Tolerated | Tolerated | Tolerated | Tolerated | Tolerated | Tolerated | Tolerated | Tolerated |
| 130. | rs748122615 | R207W | Deleterious | Tolerated | Tolerated | Tolerated | Tolerated | Tolerated | Deleterious | Deleterious |

**Table S2.** Structure based prediction of mutations associated with Bcl-2.

| **S. No.** | **Variant ID** | **Variant** | **Predictor** | | | | | | | |
| --- | --- | --- | --- | --- | --- | --- | --- | --- | --- | --- |
|  |  |  | **mCSM** | **SDM2** | **DUET** | **PremPS** | **CUPSAT** | **ENCoM** | **MutPred-2** | **DynaMut** |
| 1. | rs775404824 | G8E | Destabilizing | Destabilizing | Destabilizing | Destabilizing | Destabilizing | Stabilizing | Stabilizing | Stabilizing |
| 2. | [rs769588208](https://www.ensembl.org/Homo_sapiens/Variation/Explore?db=core;g=ENSG00000171791;r=18:63123346-63319769;t=ENST00000333681;vf=77768199) | N11S | Destabilizing | Destabilizing | Destabilizing | Destabilizing | Destabilizing | Destabilizing | Stabilizing | Stabilizing |
| 3. | rs960653284 | R12G | Destabilizing | Destabilizing | Destabilizing | Destabilizing | Stabilizing | Destabilizing | Destabilizing | Destabilizing |
| 4. | rs745851862 | V15L | Destabilizing | Stabilizing | Stabilizing | Destabilizing | Stabilizing | Stabilizing | Stabilizing | Stabilizing |
| 5. | rs776360417 | M16I | Destabilizing | Destabilizing | Destabilizing | Stabilizing | Stabilizing | Destabilizing | Stabilizing | Stabilizing |
| 6. | rs565741123 | K17N | Destabilizing | Destabilizing | Destabilizing | Destabilizing | Destabilizing | Destabilizing | Stabilizing | Destabilizing |
| 7. | rs780634396 | K17E | Destabilizing | Stabilizing | Destabilizing | Destabilizing | Destabilizing | Destabilizing | Stabilizing | Destabilizing |
| 8. | rs746600883 | I19L | Destabilizing | Stabilizing | Destabilizing | Destabilizing | Destabilizing | Stabilizing | Destabilizing | Stabilizing |
| 9. | rs758123306 | H20Q | Destabilizing | Destabilizing | Destabilizing | Destabilizing | Destabilizing | Destabilizing | Stabilizing | Destabilizing |
| 10. | rs777401949 | H20N | Destabilizing | Destabilizing | Destabilizing | Destabilizing | Destabilizing | Destabilizing | Stabilizing | Destabilizing |
| 11. | rs1441603213 | L23M | Destabilizing | Destabilizing | Destabilizing | Destabilizing | Destabilizing | Destabilizing | Stabilizing | Stabilizing |
| 12. | rs779372254 | G27S | Destabilizing | Destabilizing | Destabilizing | Destabilizing | Destabilizing | Destabilizing | Stabilizing | Destabilizing |
| 13. | rs1555711318 | Y28H | Destabilizing | Destabilizing | Destabilizing | Destabilizing | Stabilizing | Destabilizing | Stabilizing | Stabilizing |
| 14. | rs766508625 | D31H | Stabilizing | Stabilizing | Stabilizing | Stabilizing | Destabilizing | Destabilizing | Stabilizing | Destabilizing |
| 15. | rs546449806 | A32G | Destabilizing | Destabilizing | Destabilizing | Destabilizing | Stabilizing | Destabilizing | Stabilizing | Destabilizing |
| 16. | rs760840889 | A32P | Destabilizing | Destabilizing | Destabilizing | Destabilizing | Destabilizing | Destabilizing | Stabilizing | Destabilizing |
| 17. | rs767612613 | G33R | Destabilizing | Destabilizing | Destabilizing | Destabilizing | Stabilizing | Stabilizing | Stabilizing | Stabilizing |
| 18. | rs540701354 | D34Y | Stabilizing | Stabilizing | Stabilizing | Stabilizing | Destabilizing | Stabilizing | Stabilizing | Stabilizing |
| 19. | rs775108608 | V35M | Destabilizing | Destabilizing | Destabilizing | Stabilizing | Destabilizing | Destabilizing | Stabilizing | Destabilizing |
| 20. | rs1224885133 | P39L | Destabilizing | Destabilizing | Stabilizing | Destabilizing | Destabilizing | Destabilizing | Stabilizing | Destabilizing |
| 21. | rs770925898 | P40L | Destabilizing | Destabilizing | Destabilizing | Stabilizing | Destabilizing | Stabilizing | Stabilizing | Stabilizing |
| 22. | rs777309369 | G41R | Destabilizing | Destabilizing | Destabilizing | Destabilizing | Destabilizing | Stabilizing | Stabilizing | Stabilizing |
| 23. | rs1235481489 | A42T | Destabilizing | Destabilizing | Destabilizing | Stabilizing | Destabilizing | Stabilizing | Stabilizing | Stabilizing |
| 24. | rs1800477 | A43T | Destabilizing | Destabilizing | Destabilizing | Stabilizing | Stabilizing | Destabilizing | Stabilizing | Stabilizing |
| 25. | rs779096658 | A45S | Destabilizing | Destabilizing | Destabilizing | Destabilizing | Stabilizing | Stabilizing | Stabilizing | Destabilizing |
| 26. | rs755382644 | A45G | Destabilizing | Stabilizing | Stabilizing | Destabilizing | Stabilizing | Stabilizing | Stabilizing | Stabilizing |
| 27. | rs749745319 | P46A | Destabilizing | Stabilizing | Destabilizing | Destabilizing | Destabilizing | Stabilizing | Stabilizing | Stabilizing |
| 28. | rs369277721 | P46L | Destabilizing | Stabilizing | Stabilizing | Stabilizing | Destabilizing | Stabilizing | Stabilizing | Stabilizing |
| 29. | rs1448547701 | G47V | Destabilizing | Stabilizing | Stabilizing | Stabilizing | Destabilizing | Destabilizing | Stabilizing | Destabilizing |
| 30. | rs368344129 | G47S | Destabilizing | Destabilizing | Destabilizing | Stabilizing | Stabilizing | Stabilizing | Stabilizing | Stabilizing |
| 31. | rs1351068431 | S51P | Destabilizing | Stabilizing | Stabilizing | Destabilizing | Stabilizing | Destabilizing | Stabilizing | Destabilizing |
| 32. | rs1324162631 | Q52S | Destabilizing | Destabilizing | Destabilizing | Stabilizing | Destabilizing | Stabilizing | Stabilizing | Stabilizing |
| 33. | rs1245727781 | G54R | Destabilizing | Destabilizing | Destabilizing | Destabilizing | Stabilizing | Stabilizing | Stabilizing | Destabilizing |
| 34. | rs776473075 | H55Q | Stabilizing | Destabilizing | Stabilizing | Destabilizing | Destabilizing | Stabilizing | Stabilizing | Destabilizing |
| 35. | rs759325300 | H55Y | Stabilizing | Destabilizing | Stabilizing | Stabilizing | Destabilizing | Stabilizing | Stabilizing | Stabilizing |
| 36. | rs766277782 | T56R | Destabilizing | Stabilizing | Stabilizing | Destabilizing | Stabilizing | Destabilizing | Stabilizing | Stabilizing |
| 37. | rs1192476761 | P59Q | Destabilizing | Destabilizing | Destabilizing | Destabilizing | Stabilizing | Stabilizing | Stabilizing | Destabilizing |
| 38. | rs1409089747 | A60V | Destabilizing | Destabilizing | Destabilizing | Stabilizing | Destabilizing | Stabilizing | Stabilizing | Stabilizing |
| 39. | rs772554403 | A61S | Destabilizing | Destabilizing | Destabilizing | Destabilizing | Destabilizing | Stabilizing | Stabilizing | Stabilizing |
| 40. | rs747696230 | S62F | Destabilizing | Stabilizing | Destabilizing | Stabilizing | Destabilizing | Stabilizing | Stabilizing | Stabilizing |
| 41. | rs1450331336 | R63P | Stabilizing | Destabilizing | Stabilizing | Destabilizing | Stabilizing | Destabilizing | Stabilizing | Destabilizing |
| 42. | rs774056251 | R63W | Destabilizing | Stabilizing | Destabilizing | Stabilizing | Stabilizing | Stabilizing | Stabilizing | Stabilizing |
| 43. | rs1241412160 | P65L | Destabilizing | Stabilizing | Stabilizing | Stabilizing | Destabilizing | Stabilizing | Stabilizing | Stabilizing |
| 44. | rs768259110 | P65S | Destabilizing | Destabilizing | Destabilizing | Stabilizing | Destabilizing | Stabilizing | Stabilizing | Stabilizing |
| 45. | rs749649089 | V66I | Destabilizing | Stabilizing | Destabilizing | Stabilizing | Destabilizing | Stabilizing | Stabilizing | Destabilizing |
| 46. | rs756650851 | A67V | Destabilizing | Stabilizing | Stabilizing | Stabilizing | Destabilizing | Stabilizing | Stabilizing | Stabilizing |
| 47. | rs780606244 | A67S | Destabilizing | Destabilizing | Destabilizing | Stabilizing | Destabilizing | Stabilizing | Stabilizing | Stabilizing |
| 48. | rs746401408 | R68G | Destabilizing | Stabilizing | Stabilizing | Destabilizing | Destabilizing | Stabilizing | Stabilizing | Stabilizing |
| 49. | rs1338401488 | T69S | Destabilizing | Destabilizing | Destabilizing | Destabilizing | Destabilizing | Stabilizing | Stabilizing | Stabilizing |
| 50. | rs1435116050 | S70L | Destabilizing | Stabilizing | Stabilizing | Stabilizing | Destabilizing | Stabilizing | Stabilizing | Stabilizing |
| 51. | rs1486963186 | P71L | Destabilizing | Stabilizing | Stabilizing | Stabilizing | Stabilizing | Stabilizing | Stabilizing | Stabilizing |
| 52. | rs1304844332 | P71S | Destabilizing | Stabilizing | Destabilizing | Destabilizing | Destabilizing | Destabilizing | Stabilizing | Destabilizing |
| 53. | rs1299930433 | T74I | Destabilizing | Stabilizing | Stabilizing | Stabilizing | Destabilizing | Stabilizing | Stabilizing | Stabilizing |
| 54. | rs1599310991 | T74P | Destabilizing | Destabilizing | Destabilizing | Destabilizing | Destabilizing | Destabilizing | Stabilizing | Destabilizing |
| 55. | rs757360142 | P75L | Destabilizing | Stabilizing | Stabilizing | Stabilizing | Destabilizing | Stabilizing | Stabilizing | Stabilizing |
| 56. | rs1228330940 | A76T | Destabilizing | Destabilizing | Destabilizing | Stabilizing | Stabilizing | Stabilizing | Stabilizing | Destabilizing |
| 57. | rs1157110679 | A77V | Destabilizing | Destabilizing | Destabilizing | Stabilizing | Stabilizing | Stabilizing | Stabilizing | Stabilizing |
| 58. | rs753603356 | P78S | Destabilizing | Destabilizing | Stabilizing | Destabilizing | Destabilizing | Destabilizing | Stabilizing | Destabilizing |
| 59. | rs766196038 | G79R | Destabilizing | Destabilizing | Destabilizing | Stabilizing | Stabilizing | Destabilizing | Stabilizing | Destabilizing |
| 60. | rs760559670 | A80V | Destabilizing | Destabilizing | Stabilizing | Stabilizing | Stabilizing | Destabilizing | Stabilizing | Stabilizing |
| 61. | rs1477078177 | A80S | Destabilizing | Destabilizing | Destabilizing | Destabilizing | Stabilizing | Stabilizing | Stabilizing | Destabilizing |
| 62. | rs201827119 | A82V | Destabilizing | Stabilizing | Stabilizing | Stabilizing | Destabilizing | Stabilizing | Stabilizing | Destabilizing |
| 63. | rs375594294 | G83E | Destabilizing | Destabilizing | Destabilizing | Destabilizing | Destabilizing | Stabilizing | Stabilizing | Stabilizing |
| 64. | rs761382113 | P84S | Destabilizing | Destabilizing | Destabilizing | Destabilizing | Stabilizing | Stabilizing | Stabilizing | Stabilizing |
| 65. | rs770293857 | S87R | Destabilizing | Stabilizing | Destabilizing | Destabilizing | Stabilizing | Stabilizing | Stabilizing | Stabilizing |
| 66. | rs748978916 | S87G | Destabilizing | Stabilizing | Destabilizing | Destabilizing | Destabilizing | Destabilizing | Stabilizing | Destabilizing |
| 67. | rs781718615 | P88L | Destabilizing | Destabilizing | Destabilizing | Destabilizing | Stabilizing | Stabilizing | Stabilizing | Destabilizing |
| 68. | rs1467376730 | V89A | Destabilizing | Destabilizing | Destabilizing | Destabilizing | Destabilizing | Destabilizing | Stabilizing | Destabilizing |
| 69. | rs1467376730 | V93A | Destabilizing | Destabilizing | Destabilizing | Destabilizing | Destabilizing | Destabilizing | Stabilizing | Destabilizing |
| 70. | rs551395951 | H94P | Destabilizing | Destabilizing | Destabilizing | Destabilizing | Destabilizing | Destabilizing | Destabilizing | Stabilizing |
| 71. | rs373889054 | L95V | Destabilizing | Destabilizing | Destabilizing | Destabilizing | Stabilizing | Destabilizing | Stabilizing | Stabilizing |
| 72. | rs752942153 | T96A | Destabilizing | Stabilizing | Destabilizing | Stabilizing | Destabilizing | Destabilizing | Stabilizing | Stabilizing |
| 73. | rs528042823 | L97P | Destabilizing | Destabilizing | Destabilizing | Destabilizing | Destabilizing | Destabilizing | Destabilizing | Destabilizing |
| 74. | rs755908754 | R98L | Destabilizing | Stabilizing | Destabilizing | Destabilizing | Destabilizing | Destabilizing | Destabilizing | Destabilizing |
| 75. | rs559397524 | Q99R | Destabilizing | Stabilizing | Destabilizing | Destabilizing | Stabilizing | Destabilizing | Stabilizing | Stabilizing |
| 76. | rs750253286 | A100V | Destabilizing | Destabilizing | Destabilizing | Stabilizing | Destabilizing | Stabilizing | Stabilizing | Stabilizing |
| 77. | rs1184034130 | A100T | Destabilizing | Destabilizing | Destabilizing | Destabilizing | Destabilizing | Stabilizing | Stabilizing | Stabilizing |
| 78. | rs1208924867 | G101V | Destabilizing | Destabilizing | Stabilizing | Destabilizing | Stabilizing | Stabilizing | Destabilizing | Stabilizing |
| 79. | rs761696930 | D102N | Destabilizing | Stabilizing | Destabilizing | Destabilizing | Destabilizing | Destabilizing | Destabilizing | Destabilizing |
| 80. | rs1259350145 | D103Y | Destabilizing | Stabilizing | Destabilizing | Destabilizing | Stabilizing | Stabilizing | Destabilizing | Stabilizing |
| 81. | rs751038951 | F104L | Destabilizing | Stabilizing | Destabilizing | Destabilizing | Destabilizing | Destabilizing | Destabilizing | Destabilizing |
| 82. | rs762635201 | S105F | Destabilizing | Stabilizing | Destabilizing | Destabilizing | Stabilizing | Stabilizing | Stabilizing | Stabilizing |
| 83. | rs763718170 | S105P | Destabilizing | Destabilizing | Stabilizing | Destabilizing | Stabilizing | Stabilizing | Destabilizing | Stabilizing |
| 84. | rs1302520231 | R109H | Destabilizing | Stabilizing | Destabilizing | Destabilizing | Destabilizing | Stabilizing | Stabilizing | Destabilizing |
| 85. | rs1379595021 | R109C | Destabilizing | Destabilizing | Destabilizing | Destabilizing | Destabilizing | Destabilizing | Stabilizing | Stabilizing |
| 86. | rs990629198 | A113D | Destabilizing | Destabilizing | Destabilizing | Destabilizing | Stabilizing | Stabilizing | Stabilizing | Stabilizing |
| 87. | rs1284045765 | M115I | Destabilizing | Stabilizing | Stabilizing | Destabilizing | Stabilizing | Stabilizing | Stabilizing | Destabilizing |
| 88. | rs759928495 | Q118R | Stabilizing | Destabilizing | Stabilizing | Stabilizing | Stabilizing | Destabilizing | Stabilizing | Stabilizing |
| 89. | rs776905089 | L119M | Destabilizing | Destabilizing | Destabilizing | Destabilizing | Destabilizing | Stabilizing | Stabilizing | Stabilizing |
| 90. | rs373065228 | T122M | Destabilizing | Destabilizing | Destabilizing | Destabilizing | Destabilizing | Stabilizing | Stabilizing | Stabilizing |
| 91. | rs61733415 | F124L | Destabilizing | Stabilizing | Destabilizing | Destabilizing | Destabilizing | Stabilizing | Stabilizing | Stabilizing |
| 92. | rs1255855616 | R127Q | Destabilizing | Destabilizing | Destabilizing | Destabilizing | Destabilizing | Destabilizing | Stabilizing | Destabilizing |
| 93. | rs747504890 | G128R | Destabilizing | Stabilizing | Destabilizing | Stabilizing | Stabilizing | Stabilizing | Stabilizing | Stabilizing |
| 94. | rs777784952 | R129S | Destabilizing | Destabilizing | Destabilizing | Destabilizing | Destabilizing | Destabilizing | Stabilizing | Destabilizing |
| 95. | rs1239600576 | R129P | Destabilizing | Destabilizing | Destabilizing | Destabilizing | Destabilizing | Destabilizing | Destabilizing | Destabilizing |
| 96. | rs777784952 | R129C | Destabilizing | Destabilizing | Destabilizing | Destabilizing | Stabilizing | Destabilizing | Stabilizing | Destabilizing |
| 97. | rs772156175 | A131V | Destabilizing | Destabilizing | Stabilizing | Stabilizing | Destabilizing | Stabilizing | Stabilizing | Stabilizing |
| 98. | rs1368843367 | V134L | Stabilizing | Stabilizing | Stabilizing | Destabilizing | Destabilizing | Stabilizing | Stabilizing | Stabilizing |
| 99. | rs1005748945 | D140G | Destabilizing | Destabilizing | Destabilizing | Destabilizing | Destabilizing | Destabilizing | Destabilizing | Destabilizing |
| 100. | rs1473616979 | D140N | Stabilizing | Destabilizing | Stabilizing | Destabilizing | Stabilizing | Destabilizing | Destabilizing | Destabilizing |
| 101. | rs755252289 | G141E | Destabilizing | Destabilizing | Destabilizing | Destabilizing | Destabilizing | Destabilizing | Destabilizing | Destabilizing |
| 102. | rs1599310706 | V142G | Destabilizing | Destabilizing | Destabilizing | Destabilizing | Stabilizing | Destabilizing | Destabilizing | Destabilizing |
| 103. | rs1388498866 | N143S | Destabilizing | Destabilizing | Destabilizing | Destabilizing | Stabilizing | Destabilizing | Destabilizing | Stabilizing |
| 104. | rs780880487 | F150L | Destabilizing | Destabilizing | Destabilizing | Destabilizing | Destabilizing | Destabilizing | Destabilizing | Stabilizing |
| 105. | rs1446199866 | V156F | Destabilizing | Destabilizing | Destabilizing | Destabilizing | Destabilizing | Stabilizing | Destabilizing | Stabilizing |
| 106. | rs942677296 | M157L | Destabilizing | Stabilizing | Destabilizing | Stabilizing | Destabilizing | Destabilizing | Destabilizing | Stabilizing |
| 107. | rs1334020924 | V162F | Destabilizing | Destabilizing | Destabilizing | Destabilizing | Destabilizing | Stabilizing | Destabilizing | Stabilizing |
| 108. | rs1295831156 | M166I | Destabilizing | Stabilizing | Destabilizing | Destabilizing | Stabilizing | Destabilizing | Stabilizing | Destabilizing |
| 109. | rs201085318 | M166T | Destabilizing | Destabilizing | Destabilizing | Destabilizing | Stabilizing | Destabilizing | Destabilizing | Destabilizing |
| 110. | rs1219454995 | M166L | Destabilizing | Stabilizing | Destabilizing | Destabilizing | Stabilizing | Destabilizing | Stabilizing | Destabilizing |
| 111. | rs751464195 | P168S | Destabilizing | Destabilizing | Destabilizing | Destabilizing | Stabilizing | Stabilizing | Stabilizing | Destabilizing |
| 112. | rs1441924243 | N172S | Destabilizing | Destabilizing | Destabilizing | Destabilizing | Destabilizing | Stabilizing | Stabilizing | Destabilizing |
| 113. | rs909849040 | N172H | Destabilizing | Stabilizing | Destabilizing | Destabilizing | Stabilizing | Stabilizing | Stabilizing | Stabilizing |
| 114. | rs1228672103 | I173V | Destabilizing | Destabilizing | Destabilizing | Destabilizing | Destabilizing | Destabilizing | Stabilizing | Destabilizing |
| 115. | rs1178840163 | A174T | Destabilizing | Destabilizing | Destabilizing | Destabilizing | Destabilizing | Stabilizing | Destabilizing | Destabilizing |
| 116. | rs1599310597 | E179D | Destabilizing | Destabilizing | Destabilizing | Destabilizing | Destabilizing | Destabilizing | Stabilizing | Destabilizing |
| 117. | rs752310933 | L181V | Destabilizing | Destabilizing | Destabilizing | Destabilizing | Destabilizing | Destabilizing | Destabilizing | Destabilizing |
| 118. | rs1599310574 | R183Q | Stabilizing | Destabilizing | Stabilizing | Destabilizing | Stabilizing | Stabilizing | Stabilizing | Destabilizing |
| 119. | rs868435115 | R183W | Destabilizing | Stabilizing | Destabilizing | Stabilizing | Destabilizing | Stabilizing | Stabilizing | Stabilizing |
| 120. | rs1310296388 | H186R | Destabilizing | Destabilizing | Destabilizing | Destabilizing | Destabilizing | Destabilizing | Stabilizing | Destabilizing |
| 121. | rs1197820694 | G193R | Destabilizing | Destabilizing | Destabilizing | Destabilizing | Destabilizing | Stabilizing | Destabilizing | Destabilizing |
| 122. | rs1261263162 | A197D | Destabilizing | Destabilizing | Destabilizing | Destabilizing | Destabilizing | Stabilizing | Destabilizing | Destabilizing |
| 123. | rs1344438049 | A197T | Destabilizing | Destabilizing | Destabilizing | Destabilizing | Destabilizing | Stabilizing | Destabilizing | Destabilizing |
| 124. | rs1319293137 | E200D | Destabilizing | Destabilizing | Destabilizing | Destabilizing | Destabilizing | Stabilizing | Destabilizing | Destabilizing |
| 125. | rs148811059 | G203S | Destabilizing | Destabilizing | Destabilizing | Destabilizing | Destabilizing | Stabilizing | Stabilizing | Stabilizing |
| 126. | rs1380222499 | P204L | Destabilizing | Stabilizing | Stabilizing | Destabilizing | Destabilizing | Stabilizing | Stabilizing | Stabilizing |
| 127. | rs1335816980 | S205G | Destabilizing | Stabilizing | Stabilizing | Destabilizing | Destabilizing | Destabilizing | Stabilizing | Destabilizing |
| 128. | rs758367430 | M206V | Destabilizing | Stabilizing | Destabilizing | Destabilizing | Stabilizing | Destabilizing | Stabilizing | Stabilizing |
| 129. | rs369294037 | R207Q | Stabilizing | Destabilizing | Stabilizing | Destabilizing | Destabilizing | Destabilizing | Stabilizing | Stabilizing |
| 130. | rs748122615 | R207W | Destabilizing | Destabilizing | Destabilizing | Destabilizing | Destabilizing | Destabilizing | Stabilizing | Destabilizing |

**Table S3.** Pathogenicity prediction of mutations associated with Bcl-2.

| **S. No.** | **Variant ID** | **Variant** | **Predictor** | | | | |
| --- | --- | --- | --- | --- | --- | --- | --- |
|  |  |  | **Phd-SNP** | **PANTHER** | **SNPs and GO** | **PMut** | **Meta-SNP** |
| 1. | rs775404824 | G8E | Disease | Disease | Disease | Neutral | Neutral |
| 2. | [rs769588208](https://www.ensembl.org/Homo_sapiens/Variation/Explore?db=core;g=ENSG00000171791;r=18:63123346-63319769;t=ENST00000333681;vf=77768199) | N11S | Disease | Neutral | Disease | Disease | Neutral |
| 3. | rs960653284 | R12G | Disease | Disease | Disease | Disease | Disease |
| 4. | rs745851862 | V15L | Disease | Disease | Disease | Disease | Disease |
| 5. | rs776360417 | M16I | Neutral | Neutral | Neutral | Neutral | Neutral |
| 6. | rs565741123 | K17N | Neutral | Neutral | Neutral | Neutral | Neutral |
| 7. | rs780634396 | K17E | Disease | Neutral | Neutral | Neutral | Disease |
| 8. | rs746600883 | I19L | Neutral | Neutral | Neutral | Disease | Neutral |
| 9. | rs758123306 | H20Q | Neutral | Disease | Neutral | Neutral | Neutral |
| 10. | rs777401949 | H20N | Neutral | Disease | Neutral | Disease | Neutral |
| 11. | rs1441603213 | L23M | Disease | Disease | Disease | Disease | Neutral |
| 12. | rs779372254 | G27S | Disease | Neutral | Disease | Disease | Neutral |
| 13. | rs1555711318 | Y28H | Disease | Disease | Neutral | Disease | Neutral |
| 14. | rs766508625 | D31H | Neutral | Neutral | Neutral | Neutral | Neutral |
| 15. | rs546449806 | A32G | Neutral | Neutral | Neutral | Neutral | Neutral |
| 16. | rs760840889 | A32P | Neutral | Neutral | Neutral | Neutral | Neutral |
| 17. | rs767612613 | G33R | Neutral | Disease | Neutral | Neutral | Neutral |
| 18. | rs540701354 | D34Y | Neutral | Disease | Neutral | Neutral | Neutral |
| 19. | rs775108608 | V35M | Neutral | Neutral | Neutral | Neutral | Neutral |
| 20. | rs1224885133 | P39L | Neutral | Neutral | Neutral | Neutral | Neutral |
| 21. | rs770925898 | P40L | Neutral | Neutral | Neutral | Neutral | Neutral |
| 22. | rs777309369 | G41R | Neutral | Neutral | Neutral | Neutral | Neutral |
| 23. | rs1235481489 | A42T | Neutral | Neutral | Neutral | Neutral | Neutral |
| 24. | rs1800477 | A43T | Neutral | Neutral | Neutral | Neutral | Neutral |
| 25. | rs779096658 | A45S | Neutral | Neutral | Neutral | Neutral | Neutral |
| 26. | rs755382644 | A45G | Neutral | Neutral | Neutral | Neutral | Neutral |
| 27. | rs749745319 | P46A | Neutral | Neutral | Neutral | Neutral | Neutral |
| 28. | rs369277721 | P46L | Neutral | Disease | Neutral | Neutral | Neutral |
| 29. | rs1448547701 | G47V | Neutral | Neutral | Neutral | Neutral | Neutral |
| 30. | rs368344129 | G47S | Neutral | Neutral | Neutral | Neutral | Neutral |
| 31. | rs1351068431 | S51P | Neutral | Neutral | Neutral | Neutral | Neutral |
| 32. | rs1324162631 | Q52S | Neutral | Neutral | Neutral | Neutral | Neutral |
| 33. | rs1245727781 | G54R | Neutral | Neutral | Neutral | Neutral | Neutral |
| 34. | rs776473075 | H55Q | Neutral | Neutral | Neutral | Neutral | Neutral |
| 35. | rs759325300 | H55Y | Neutral | Neutral | Neutral | Neutral | Neutral |
| 36. | rs766277782 | T56R | Neutral | Neutral | Neutral | Neutral | Neutral |
| 37. | rs1192476761 | P59Q | Neutral | Disease | Neutral | Neutral | Neutral |
| 38. | rs1409089747 | A60V | Neutral | Neutral | Neutral | Neutral | Neutral |
| 39. | rs772554403 | A61S | Neutral | Neutral | Neutral | Neutral | Neutral |
| 40. | rs747696230 | S62F | Neutral | Neutral | Neutral | Neutral | Neutral |
| 41. | rs1450331336 | R63P | Neutral | Neutral | Neutral | Neutral | Neutral |
| 42. | rs774056251 | R63W | Neutral | Disease | Neutral | Neutral | Neutral |
| 43. | rs1241412160 | P65L | Neutral | Neutral | Neutral | Neutral | Neutral |
| 44. | rs768259110 | P65S | Neutral | Neutral | Neutral | Neutral | Neutral |
| 45. | rs749649089 | V66I | Neutral | Neutral | Neutral | Neutral | Neutral |
| 46. | rs756650851 | A67V | Neutral | Neutral | Neutral | Neutral | Neutral |
| 47. | rs780606244 | A67S | Neutral | Neutral | Neutral | Neutral | Neutral |
| 48. | rs746401408 | R68G | Neutral | Neutral | Neutral | Neutral | Neutral |
| 49. | rs1338401488 | T69S | Neutral | Neutral | Neutral | Neutral | Neutral |
| 50. | rs1435116050 | S70L | Neutral | Neutral | Neutral | Neutral | Neutral |
| 51. | rs1486963186 | P71L | Neutral | Neutral | Neutral | Neutral | Neutral |
| 52. | rs1304844332 | P71S | Neutral | Neutral | Neutral | Neutral | Neutral |
| 53. | rs1299930433 | T74I | Neutral | Neutral | Neutral | Neutral | Neutral |
| 54. | rs1599310991 | T74P | Neutral | Neutral | Neutral | Neutral | Neutral |
| 55. | rs757360142 | P75L | Neutral | Neutral | Neutral | Neutral | Neutral |
| 56. | rs1228330940 | A76T | Neutral | Neutral | Neutral | Neutral | Neutral |
| 57. | rs1157110679 | A77V | Neutral | Neutral | Neutral | Neutral | Neutral |
| 58. | rs753603356 | P78S | Neutral | Neutral | Neutral | Neutral | Neutral |
| 59. | rs766196038 | G79R | Neutral | Neutral | Neutral | Neutral | Neutral |
| 60. | rs760559670 | A80V | Neutral | Neutral | Neutral | Neutral | Neutral |
| 61. | rs1477078177 | A80S | Neutral | Neutral | Neutral | Neutral | Neutral |
| 62. | rs201827119 | A82V | Neutral | Neutral | Neutral | Neutral | Neutral |
| 63. | rs375594294 | G83E | Neutral | Neutral | Neutral | Neutral | Neutral |
| 64. | rs761382113 | P84S | Neutral | Disease | Neutral | Neutral | Neutral |
| 65. | rs770293857 | S87R | Neutral | Neutral | Neutral | Neutral | Neutral |
| 66. | rs748978916 | S87G | Neutral | Neutral | Neutral | Neutral | Neutral |
| 67. | rs781718615 | P88L | Neutral | Disease | Neutral | Neutral | Neutral |
| 68. | rs1467376730 | V89A | Neutral | Neutral | Neutral | Neutral | Neutral |
| 69. | rs1467376730 | V93A | Neutral | Neutral | Neutral | Disease | Neutral |
| 70. | rs551395951 | H94P | Disease | Disease | Disease | Disease | Disease |
| 71. | rs373889054 | L95V | Neutral | Neutral | Neutral | Neutral | Neutral |
| 72. | rs752942153 | T96A | Neutral | Neutral | Neutral | Neutral | Neutral |
| 73. | rs528042823 | L97P | Disease | Disease | Disease | Disease | Disease |
| 74. | rs755908754 | R98L | Disease | Disease | Disease | Disease | Disease |
| 75. | rs559397524 | Q99R | Neutral | Neutral | Neutral | Neutral | Neutral |
| 76. | rs750253286 | A100V | Neutral | Neutral | Neutral | Disease | Neutral |
| 77. | rs1184034130 | A100T | Neutral | Neutral | Neutral | Disease | Neutral |
| 78. | rs1208924867 | G101V | Disease | Disease | Neutral | Disease | Disease |
| 79. | rs761696930 | D102N | Neutral | Neutral | Neutral | Disease | Neutral |
| 80. | rs1259350145 | D103Y | Disease | Disease | Neutral | Neutral | Disease |
| 81. | rs751038951 | F104L | Neutral | Neutral | Neutral | Disease | Disease |
| 82. | rs762635201 | S105F | Neutral | Disease | Neutral | Disease | Disease |
| 83. | rs763718170 | S105P | Disease | Disease | Neutral | Neutral | Neutral |
| 84. | rs1302520231 | R109H | Neutral | Neutral | Neutral | Neutral | Neutral |
| 85. | rs1379595021 | R109C | Neutral | Disease | Neutral | Disease | Disease |
| 86. | rs990629198 | A113D | Neutral | Disease | Neutral | Neutral | Neutral |
| 87. | rs1284045765 | M115I | Neutral | Neutral | Neutral | Neutral | Neutral |
| 88. | rs759928495 | Q118R | Neutral | Disease | Neutral | Disease | Neutral |
| 89. | rs776905089 | L119M | Neutral | Neutral | Neutral | Disease | Neutral |
| 90. | rs373065228 | T122M | Neutral | Disease | Disease | Disease | Disease |
| 91. | rs61733415 | F124L | Neutral | Neutral | Neutral | Neutral | Neutral |
| 92. | rs1255855616 | R127Q | Neutral | Neutral | Neutral | Disease | Neutral |
| 93. | rs747504890 | G128R | Neutral | Neutral | Neutral | Neutral | Neutral |
| 94. | rs777784952 | R129S | Neutral | Neutral | Neutral | Neutral | Neutral |
| 95. | rs1239600576 | R129P | Disease | Disease | Disease | Disease | Disease |
| 96. | rs777784952 | R129C | Disease | Disease | Neutral | Neutral | Disease |
| 97. | rs772156175 | A131V | Neutral | Neutral | Neutral | Neutral | Neutral |
| 98. | rs1368843367 | V134L | Neutral | Neutral | Neutral | Neutral | Neutral |
| 99. | rs1005748945 | D140G | Disease | Neutral | Disease | Disease | Disease |
| 100. | rs1473616979 | D140N | Disease | Neutral | Neutral | Disease | Disease |
| 101. | rs755252289 | G141E | Disease | Disease | Disease | Disease | Disease |
| 102. | rs1599310706 | V142G | Disease | Disease | Disease | Disease | Disease |
| 103. | rs1388498866 | N143S | Disease | Disease | Disease | Disease | Disease |
| 104. | rs780880487 | F150L | Neutral | Neutral | Neutral | Neutral | Neutral |
| 105. | rs1446199866 | V156F | Neutral | Disease | Neutral | Disease | Neutral |
| 106. | rs942677296 | M157L | Neutral | Neutral | Neutral | Disease | Neutral |
| 107. | rs1334020924 | V162F | Disease | Disease | Neutral | Disease | Neutral |
| 108. | rs1295831156 | M166I | Disease | Disease | Neutral | Disease | Neutral |
| 109. | rs201085318 | M166T | Disease | Disease | Disease | Disease | Disease |
| 110. | rs1219454995 | M166L | Neutral | Disease | Neutral | Disease | Neutral |
| 111. | rs751464195 | P168S | Neutral | Disease | Neutral | Neutral | Neutral |
| 112. | rs1441924243 | N172S | Neutral | Neutral | Neutral | Neutral | Neutral |
| 113. | rs909849040 | N172H | Neutral | Neutral | Neutral | Neutral | Neutral |
| 114. | rs1228672103 | I173V | Neutral | Neutral | Neutral | Disease | Neutral |
| 115. | rs1178840163 | A174T | Neutral | Neutral | Neutral | Disease | Neutral |
| 116. | rs1599310597 | E179D | Neutral | Neutral | Neutral | Neutral | Neutral |
| 117. | rs752310933 | L181V | Neutral | Disease | Disease | Disease | Disease |
| 118. | rs1599310574 | R183Q | Neutral | Neutral | Neutral | Disease | Neutral |
| 119. | rs868435115 | R183W | Neutral | Disease | Neutral | Disease | Disease |
| 120. | rs1310296388 | H186R | Neutral | Neutral | Disease | Neutral | Neutral |
| 121. | rs1197820694 | G193R | Disease | Disease | Disease | Disease | Disease |
| 122. | rs1261263162 | A197D | Disease | Neutral | Disease | Disease | Neutral |
| 123. | rs1344438049 | A197T | Neutral | Neutral | Neutral | Neutral | Neutral |
| 124. | rs1319293137 | E200D | Neutral | Neutral | Neutral | Neutral | Neutral |
| 125. | rs148811059 | G203S | Disease | Neutral | Neutral | Neutral | Neutral |
| 126. | rs1380222499 | P204L | Neutral | Neutral | Neutral | Neutral | Neutral |
| 127. | rs1335816980 | S205G | Neutral | Neutral | Neutral | Neutral | Neutral |
| 128. | rs758367430 | M206V | Neutral | Neutral | Neutral | Neutral | Neutral |
| 129. | rs369294037 | R207Q | Neutral | Neutral | Neutral | Neutral | Neutral |
| 130. | rs748122615 | R207W | Neutral | Disease | Neutral | Disease | Neutral |

**
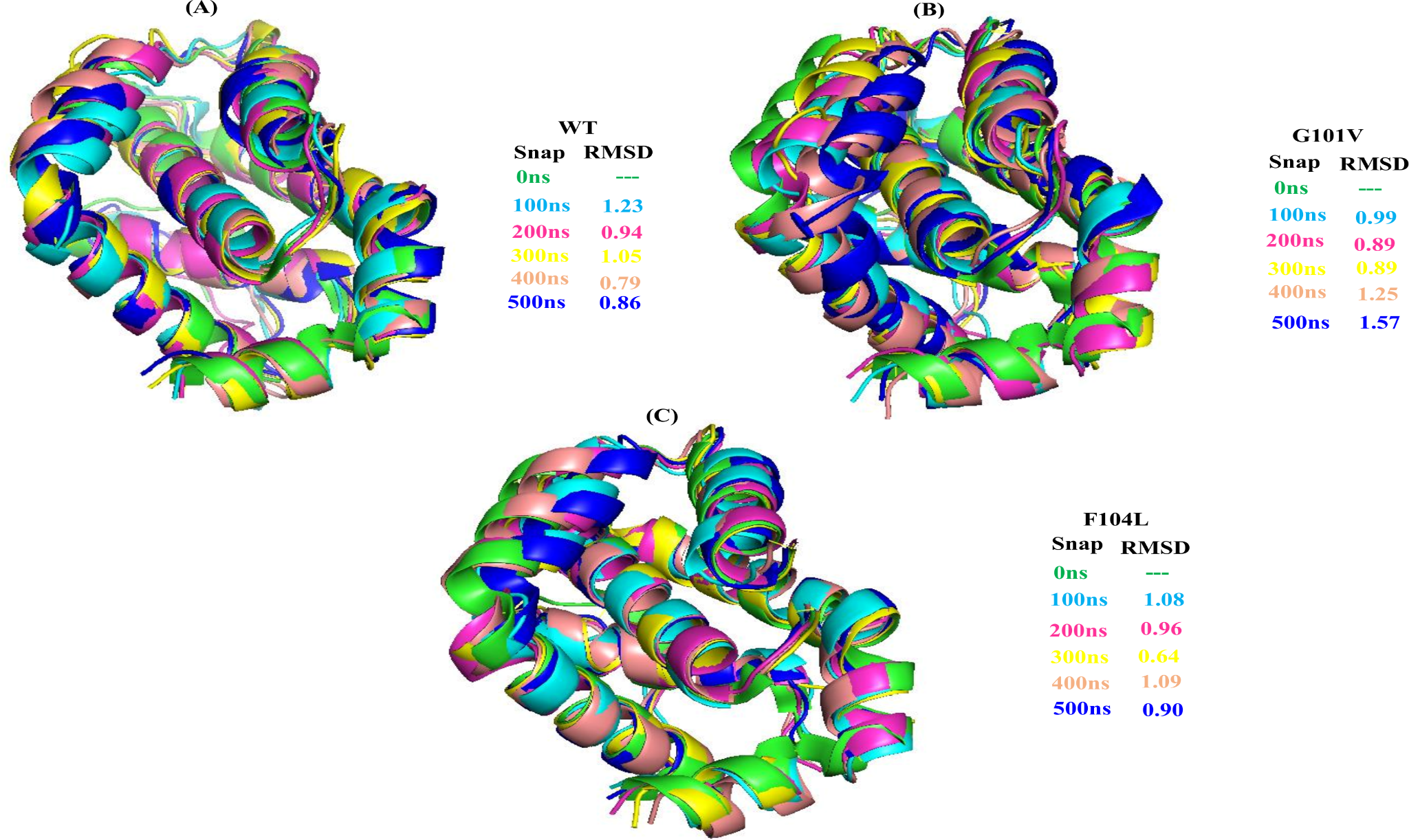
**

**Figure S1:** Structural snapshots of Bcl-2 (A) WT, (B) G101V, and (C) F104L at an interval of 100 ns from 0 to 500 ns of simulation. Corresponding panels show the RMSD of structural alignment of Bcl-2 at different time. Structures were drawn using PyMOL (<https://pymol.org/2>).
